# In What Ways Do Synthetic Nucleotides and Natural Base Lesions Alter the Structural Stability of G-Quadruplex Nucleic Acids?

**DOI:** 10.1155/2017/1641845

**Published:** 2017-10-18

**Authors:** Janos Sagi

**Affiliations:** Rimstone Laboratory, RLI, Carlsbad, CA 92010, USA

## Abstract

Synthetic analogs of natural nucleotides have long been utilized for structural studies of canonical and noncanonical nucleic acids, including the extensively investigated polymorphic G-quadruplexes (GQs). Dependence on the sequence and nucleotide modifications of the folding landscape of GQs has been reviewed by several recent studies. Here, an overview is compiled on the thermodynamic stability of the modified GQ folds and on how the stereochemical preferences of more than 70 synthetic and natural derivatives of nucleotides substituting for natural ones determine the stability as well as the conformation. Groups of nucleotide analogs only stabilize or only destabilize the GQ, while the majority of analogs alter the GQ stability in both ways. This depends on the preferred* syn* or* anti* N-glycosidic linkage of the modified building blocks, the position of substitution, and the folding architecture of the native GQ. Natural base lesions and epigenetic modifications of GQs explored so far also stabilize or destabilize the GQ assemblies. Learning the effect of synthetic nucleotide analogs on the stability of GQs can assist in engineering a required stable GQ topology, and exploring the* in vitro* action of the single and clustered natural base damage on GQ architectures may provide indications for the cellular events.

## 1. Introduction

In the course of the last two decades, substantial worldwide research activity has been devoted to the elucidation of the structural and biochemical properties of the non-B, polymorphic GQ structures, both* in vitro* and* in vivo*. Many laboratories used synthetic nucleotide building blocks, which were reviewed by several publications [1–5]. Studies have also focused on such site-specific incorporations of nucleotide analogs that could prompt the modified oligonucleotides to fold into specific GQ topologies, as summarized in [6–8]. In addition to folding topology, the fundamental property of GQ structures is their thermodynamic stability. Multiple aspects of the effect of mutation by natural nucleotides on GQ stability are well known [2–5, 9–11]. Properties of GQs built from peptide nucleic acid (PNA) units [12, 13] and of a few other special modifications, such as the (R)-1-O-(pyren-1-ylmethyl)glycerol (intercalating nucleic acid, INA) or (R)-1-O-[4-(1-pyrenylethynyl)-phenylmethyl]glycerol (twisted intercalating nucleic acid, TINA) have also been analyzed [4, 14]; therefore, these modifications are not discussed here. Much less has been summed up on the effect of synthetic constituents on the thermal and thermodynamic stability of the modified GQs, which is thus the subject of the present review. Other important current topics are the effect of single and multiple natural base lesions on GQ stability as well as the epigenetic modifications occurring in GQs, the present status of which is also reviewed here up to midsummer 2017.

**Figure 1:**
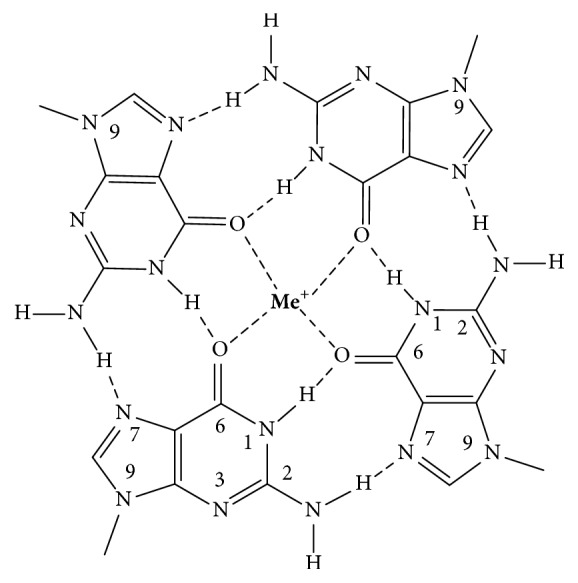
Schematic representation of a G-tetrad held together by Hoogsteen-type circular double hydrogen bonds and the metal ion.

**Figure 2:**
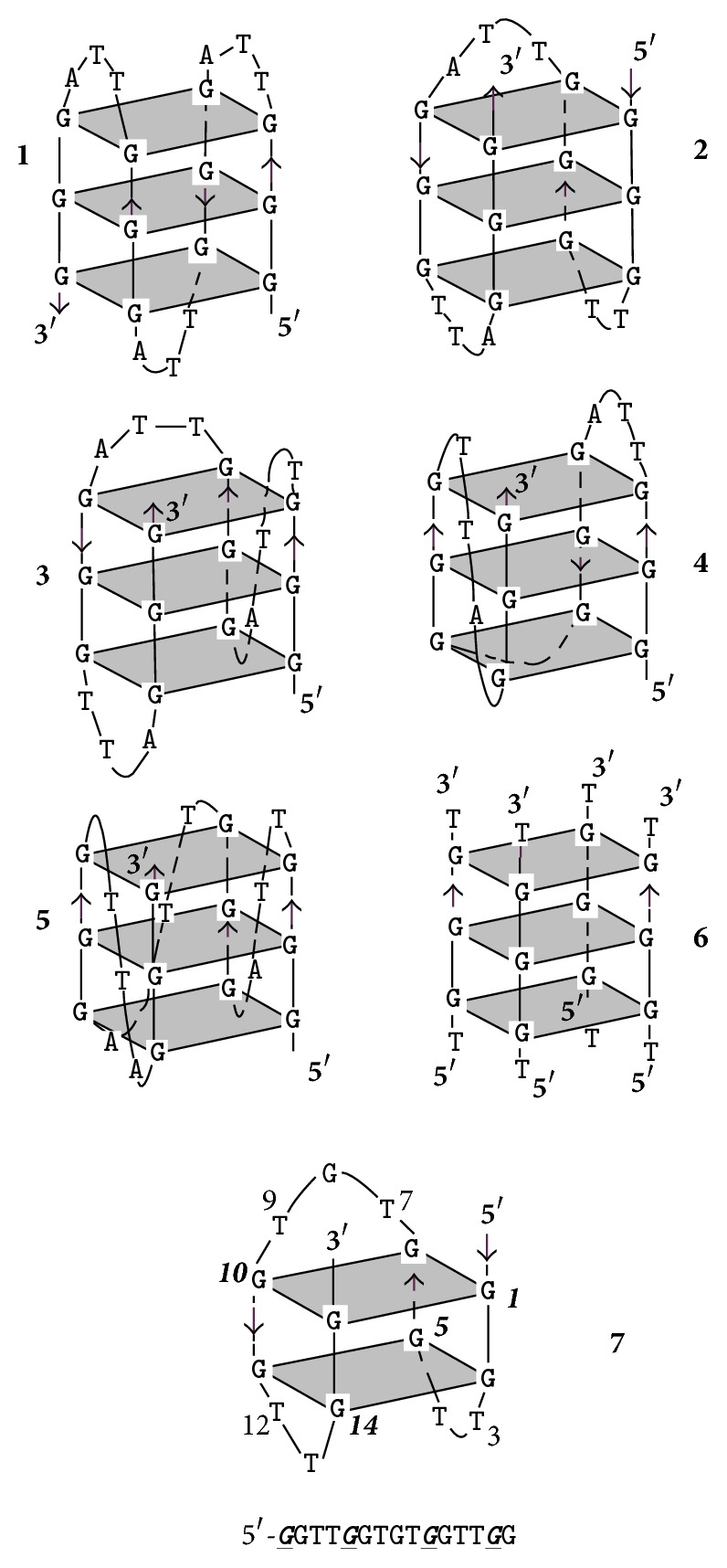
Folding arrangements of the most often cited GQ structures. The intramolecular GQ architectures adopted by the 21-mer htel-21 G_3_(TTAG_3_)_3_ oligodeoxynucleotide under various solution conditions, topologies** 1**–**5**. The basket-type antiparallel with 5′-to-3′ edgewise-diagonal-edgewise loops, forming in Na^+^ (topology** 1**); the chair-type antiparallel with three edgewise loops (topology** 2**); the (3 + 1 strands) hybrid-1 with propeller-like, edgewise, and edgewise loops (topology** 3**); the hybrid-2 with two edgewise loops followed by a propeller-type loop (topology** 4**), both hybrids forming in K^+^ solution; the parallel topology with all three loops in propeller-like configuration, as found in crystals containing K^+^ ions (topology** 5**); tetramolecular GQ assembling from four TGGGT DNA chains, [TG_3_T]_4_ (topology** 6**). Bottom panel: the two-tetrad, chair-type intramolecular antiparallel topology, folding from the 15-mer TBA oligodeoxynucleotide sequence shown below the topology. The* syn* dG nucleotides are indicated with underlined letters in italics. For references, see, for example, [8]. Shaded squares represent the G-tetrads displayed in Figure 1.

**Figure 3:**
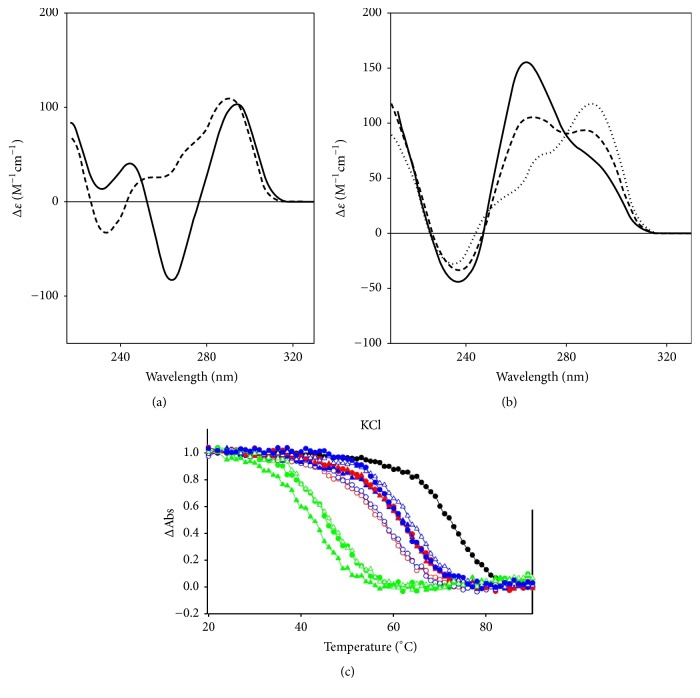
CD spectra of different GQ topologies and a series of thermal unfolding profiles. (a) shows the CD spectra of the antiparallel basket architecture of htel-21 GQ recorded in 100 mM Na^+^ solution and characterized by positive Cotton effects near 295 nm and 240 nm and a negative one around 260 nm (solid line), as well as the polymorphic form adopted by the same oligodeoxynucleotide in 100 mM K^+^, which is a mixture of K^+^-stabilized antiparallel and the hybrid forms, with the positive ellipticity near 290 nm, a strong shoulder around 270 nm, and a negative one close to 240 nm (broken line). (b) displays, for comparison, the CD spectra of the same K^+^-stabilized mixture of the htel-21 GQ (dotted line) and the mixture of just the hybrid forms with two positive peaks of close to equal heights at 290 and 260 nm (dashed line), all determined at low (50–100 *μ*M) DNA strand concentrations, as well as the majority parallel forms adopted at/above 2 mM of the strands (solid line) (see references in [8]). (c) illustrates normalized thermal unfolding profiles of 8-oxoguanine containing htel-21 GQs [26].

Single or double-helical guanine-rich (G-rich) natural or synthetic DNA and RNA sequences can, under appropriate physical (cations, cosolvents) or cellular (e.g., negative superhelical stress) conditions, convert into hairpin, triple-helical, and four-stranded GQ structures. A GQ comprises two or more G-tetrads, which are connected by loops of one or more nucleotides. The planar G-tetrads are formed through Hoogsteen-type circular double hydrogen bonds (Figure 1), and the cations are located either in the central cavity of the G-tetrad or in the spaces between the tetrads. The GQ fold is held together by the stacking of G-tetrads and the cation coordination via the O6 carbonyl atoms of the guanines. In general, the GQs are polymorphic, can be built from single or multiple molecules, and can contain antiparallel, parallel strand, or mixed (hybrid) orientations, and these features result in distinct topological arrangements. Schematic folding architectures of GQs assembling from the 21-nucleotide-long (21-mer) basic human telomere (htel) repeat DNA sequence, htel-21, and of the 15-mer thrombin-binding aptamer (TBA) oligodeoxynucleotide are shown in Figure 2. Characteristic CD spectra of the three main forms of the htel GQs are illustrated in Figure 3. The TBA GQ is the most frequently cited structure in this review due to the dozens of studies performed worldwide using this 15-mer DNA model (e.g., [3, 7, 10, 15–17]). The advantage of it is the well-defined stable and single folding topology, which contrasts the polymorphic nature of most other intramolecular GQs known, especially those built from the TTAGGG htel repeat sequences [8]. In physiological K^+^ concentrations, the TBA sequence forms a two-tetrad, chair-type intramolecular antiparallel structure with three edge-type loops, two TT, and one TGT loop (Figure 2). Positions of T4, T9, T13, and G8 are supposed to have rigid positions critical for the stability, and the T3, T7, and T12 are more flexible, not stacked to residues of the G-tetrads [18–20]. TBA GQ inhibits the thrombin-catalyzed fibrin-clot formation with a *K*_*i*_ of approximately 120 nM by binding to the thrombin protein with high selectivity. Thrombin is a serine protease that converts soluble fibrinogen to fibrin, thus stimulating platelet aggregation, that is, the clotting process (see references in [21]).

The effect of the synthetic nucleotide analogs is discussed here according to their stabilizing and/or destabilizing action. Those, around a dozen analogs that, under the specific conditions studied, were found to only stabilize or not affect the stability beyond the experimental error of determination of a specific GQ fold, make up one group of the analogs. Majority of the synthetic nucleotides studied so far that either stabilize or destabilize the GQ constitute another, the largest group, and those analogs that only destabilized a GQ belong to the third group of synthetic nucleotide analogs. Similarly, the natural base lesions were also grouped according to their stabilizing or destabilizing effects.

Stability of a GQ structure can be most properly characterized by its thermodynamic parameters communicated by the free energy change value, Δ*G*°, at various temperatures, such as Δ*G*°_37_, in kcal/mol. Thermodynamic parameters can be determined by the model-dependent method, such as the “two-state” method using temperature-dependent melting profiles by UV or CD absorption spectroscopy [22, 23], or, more exactly, by the model-independent differential scanning calorimetry (DSC) method [24, 25]. DSC is certainly the more accurate method as it explores the details of the thermal transition contrary to the integrating absorption methods (although not all folding-unfolding transitions fit the two-state requirement, several laboratories neglect it and use the two-state absorption method). Both methods provide the half-way thermal transition temperature, *T*_*m*_ or* T*_1/2_.* T*_1/2_ has been used when the annealing (refolding) is slower than the unfolding, which results in hysteresis of the thermal profile. Since most studies do not publish free energy change values but all publish *T*_*m*_ or* T*_1/2_ values, the best way to compare the stabilities of various GQ structures is to use the thermal stability values. Δ*G*° values will be mentioned herein in selected cases. Figure 3 shows typical thermal melting profiles as determined by UV absorption spectroscopy at 296 nm [26].

## 2. Synthetic Nucleotide Analogs That Stabilized or Barely Impacted the Stability of a GQ

Few synthetic analogs of natural nucleotides are currently known, which only stabilize a GQ independently of their* syn* or* anti* isomeric conformation and/or the position of the nucleotide they replaced in the potential GQ-forming oligonucleotide. Similarly, a limited number of analogs have so far been described that do not affect the thermodynamic stability of GQs beyond the experimental error of determination. It is important to note that these effects were observed in a given GQ system; the nucleotide analogs may induce the opposite effect in other, so far not-studied GQs.

**Figure 4:**
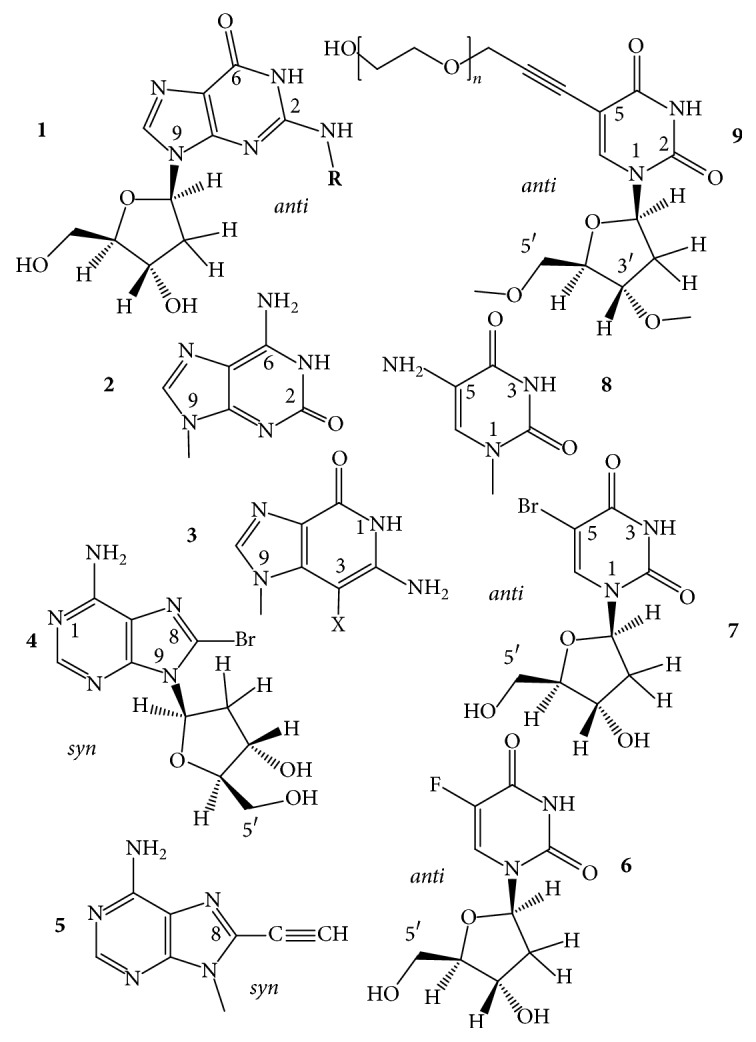
Structures of the* anti* N2-substituted-dG (**1**); isoguanine (**2**); 3-halo-3-deazaguanine (**3**);* syn* 8-bromo-dA (**4**); 8-propynyladenine (**5**);* anti* 5-fluoro-dU (**6**);* anti* 5-bromo-dU (**7**); 5-aminouracil (**8**); and oligo(ethylene glycol) substituted* anti* 5-propynyl-dU (**9**).

*N2-Guanine Derivatives*. The C2-amino group of dG (Figure 4,** 1**) is one of two positions of dG whose substitutions do not interfere with the circular double H-bonding of a G-tetrad [27, 28]. Substitution of N2 does not change the glycosidic torsion angle of the* anti* dG. The 2-amino group of guanine substituted with hydrophobic groups can enhance the thermodynamic stability of a GQ via disrupting the water sphere around the core. Water depletion is known to stabilize a GQ (and destabilize duplex DNAs) [29, 30]. The TGGGAG oligonucleotide DNA modified at the 3′- and 5′-ends with 3,4-dibenzyloxybenzyl groups (R-95288, Hotoda oligonucleotide), which forms a parallel tetramolecular GQ (Figure 2,** 6**), has anti-HIV-1 activity through binding to gp120 [31]. dGs of this oligodeoxynucleotide had been substituted with N2-methyl-2′-deoxyguanosine (m^2^dG). The m^2^dG modifications enhanced the stability of the parent GQ and the modified oligonucleotides showed a 2-fold higher anti-HIV activity than the R-95288. The GQs formed from T$\underline{m^{2}G}$GGAG and TGG$\underline{m^{2}G}$AG had higher *T*_*m*_ values, 70°C, than that assembled from TGGGAG, *T*_*m*_ of 60°C. On the other hand, the 3′-terminal substitution with m^2^dG had no effect on the GQ stability due to the poor stacking of the terminal base. The increased stabilities were explained by improved base stacking originating from the methylation of NH_2_ of G2. The GQ of T[$\underline{m^{2}G}$]_3_AG with three contiguous m^2^dG residues had the highest *T*_*m*_, which was over 80°C. The alkylation of N2G seems to be a promising way to improve the stability of parallel GQs, as long as the modification does not sterically hinder the formation of the GQs [32]. Substitution of N2 of G was also mentioned in connection with the TBA GQ (Figure 2,** 7**), however, only in connection with the thrombin-binding activity: attaching of a benzyl group to N2 of the* anti *N-glycosidic dGs (Figure 4,** 1**) of positions G6 and G11 and a 1-naphthylmethyl group into the N2 of G6 of the TBA increased the thrombin inhibitory activity. The 1-naphthylmethyl group substitution at the N2 of G6 showed about a 60% increase in activity both* in vitro* and* in vivo*. Substitutions on N2 of other G residues of the sequence had a little effect or decreased the activity [33]. The latest example for stabilization of GQ structures by N2-dG substitutions comes from using htel GQs. Lech and Phan [34] reported on the incorporation of N2-methyl, N2-hexylamino, and the N2-benzyl derivatives of dG into an htel-24 DNA GQ, the TT(htel-21)A. The substitutions stabilized the G-quadruplex by 2 to 13°C per modification.

*Isoguanine (iG)*. 2-Oxo-6-aminopurine, isoguanine (Figure 4,** 2**), has been thoroughly studied in GQ models, first by Seela and coworkers [35] with T_4_**iG**_4_T_4_ that formed a tetramolecular parallel GQ, which contained iG-tetrads held together by circular double hydrogen bonds. The [T_4_**iG**_4_T_4_]_4_ proved to be a more stable structure than the parent [T_4_G_4_T_4_]_4_ in buffered 1 M NaCl, 10 mM MgCl_2_, pH 7 [36]. The T_8_**iG**_4_T also formed parallel GQ with iG-tetrads [37]. The iG formed not only tetrads but also pentads, giving rise to pentaplexes, such as [T_4_**iG**_4_T]_5_, and the pentad planes were held together, as the tetrads, by circular double H-bonds [38–40]. The iG-pentaplexes were found to bind and activate the Fe(III)-heme complex towards peroxidase activity, comparably to GQs and contrary to iGQs [41]. In the intramolecular GQ of TBA, a single iG residue was found to enhance the binding of GQ to the human R-thrombin, as compared to the parent GQ aptamer. The most active aptamer was built from GGTTGGT**iG**TGGTTGG, and its approximate binding enhancement was 2-fold over the unmodified aptamer. The authors supposed that iG-GQs tended to form and denature more easily than the corresponding structures bearing G. Whether this would mean increased or decreased thermodynamic stability is not known [21].

*3-Deazaguanine (c* ^3^ *G)*. Quantum chemical methods-based models showed that 3-halo-3-deazaguanines (Figure 4,** 3**) replacing guanines in two- or three-tetrad GQs result in substantial energy gain leading to elevated stability for the GQs, which mainly originates from increased *π*-*π* stacking interactions [42].

*8-Bromo-, 8-Methyl-, and 8-Propynyladenines*. In the tetramolecular GQ-forming TAGGGT and AGGGT oligodeoxynucleotides, the adenines have been replaced by 8-bromadenine (br^8^dA), 8-propynyladenine (py^8^dA) (Figure 4,** 4**,** 5**) [43–45], and 8-methyladenine (m^8^A) [46, 47]. All these A-modified oligonucleotides formed the same parallel-type tetramolecular GQs as the two unmodified oligonucleotides did. While py^8^A decreased the stability of duplex DNAs, it markedly elevated the thermal stability of the tetramolecular structures, assumingly, due to a prevalent glycosidic* syn *conformation [43, 45]. br^8^A also increased the stability of the parent structures [44]. The GQ analogs of TAGGGT did not contain T, A, or 8-substituted-A tetrads, whereas the GQ of AGGGT formed an A-tetrad at the 5′-end. An m^8^dA tetrad was also formed; it was an all-*syn *m^8^dA tetrad that had only a minor effect on *T*_*m*_ of the parent structure.

*5-Fluorouracil (fl* ^5^ *U)*. Thymines of the TBA oligodeoxynucleotide were replaced, one by one, with 5-fluoro-2′-deoxyuridines (Figure 4,** 6**) by Virgilio et al. [48]. The 5-fluoro substitution for the 5-methyl group elevated the thermal stability of the GQ in each of the six cases studied, mostly by 1–3°C; however, in positions 4 and 13, fl^5^U enhanced the stability by 11 and 10°C, respectively. These two GQs also showed improved anticoagulant activity. Thymine bases at the T4 and T13 loop positions stack to the tetrad residues [18–20]. The high *T*_*m*_ values may indicate that the fluoro atom enhanced the strength of stacking. Thymines in other loop positions do not interact with the GQ core. The modified GQs adopted the same type of antiparallel topology as the wild-type did, based on the CD spectra.

*5-Bromo-, 5-Amino-, and 5-Hydroxyuracils*. Tetrad thymines had been replaced by the synthetic 5-bromouracil (br^5^U) and 5-aminouracil (n^5^U) (Figure 4,** 7**,** 8**) that substituted for the central T of TGG**T**GGT. The oligonucleotides modified by these dU-analogs formed tetramolecular parallel GQs, as the unmodified did, with all residues having* anti* glycosidic conformation. Order of thermal stability was n^5^U > br^5^U > T-containing GQs. The [TGG$\underline{n^{5}U}$GGT]_4_ GQ showed a biphasic melting profile, which was explained by noncooperative melting. The n^5^dU tetrad was observed having circular double H-bonds and this provided higher stability than T or br^5^U [49]. Using Density Functional Theory calculations and ESI-MS measurements, Paragi et al. [50] hypothesized that 5-amino- and especially 5-hydroxyuracils can form full pyrimidine and also mixed tetrads with guanine or xanthine, where tetrads have similar energy and cover almost exactly the same area as the full guanine or xanthine tetrads.

*5-Propynyluracil and Its OEG and PEG Derivatives*. Based on their previous findings [51] Tateishi-Karimata and coworkers [52] described in a recent paper the stabilizing effect of 5-propynyl-2′-deoxyuridine on the TBA GQ when it was substituted for T4 and T7 of the loops. T base at position 4 stacks to the G-tetrad, while the T7 does not [18–20]. The propynyluracil substitution of T4 enhanced the stability of the native TBA GQ (*T*_*m*_: 50.7°C, Δ*G*°_25_:  −3.5 kcal/mol) up to 61.7°C and −5.4 kcal/mol, whereas the nonstacking did not change it: 50.9°C and −3.6 kcal/mol, in buffered 100 mM KCl. The propynyluracil was further derivatized with ethylene glycols (Figure 4,** 9**). Oligo- and polyethylene glycols (OEG and PEG) are used as crowding agents* in vitro* to mimic the intracellular environments. Both destabilize the dsDNA but stabilize the GQs [30]. Various lengths and numbers of ethylene glycols were attached to the 5-(1-propynyl)-tethered dUs and substituted for the T4, T7, and T13 of the TBA GQ to learn whether these glycols interact with the GQ. Based on thermodynamic and NMR spectral analyses, they have found that the glycols do interact with the bases of the GQ, and the octaethylene (OEG8) and longer-chain glycols elevated the thermodynamic stability of the parent GQ beyond the stability increase induced by the propynyl tether alone. For instance, the *T*_*m*_ and Δ*G*°_25_ values of 50.7°C and −3.5 kcal/mol for the TBA GQ were increased to 64.3°C and −7.1 kcal/mol and 55.2°C and −4.4 kcal/mol for the propynyl-OEG(8)-dU at positions 4 and 7, respectively. The results suggested that PEG molecules interact with tetrad and loop bases via CH-*π* and lone pair-*π* interactions [52].

**Figure 5:**
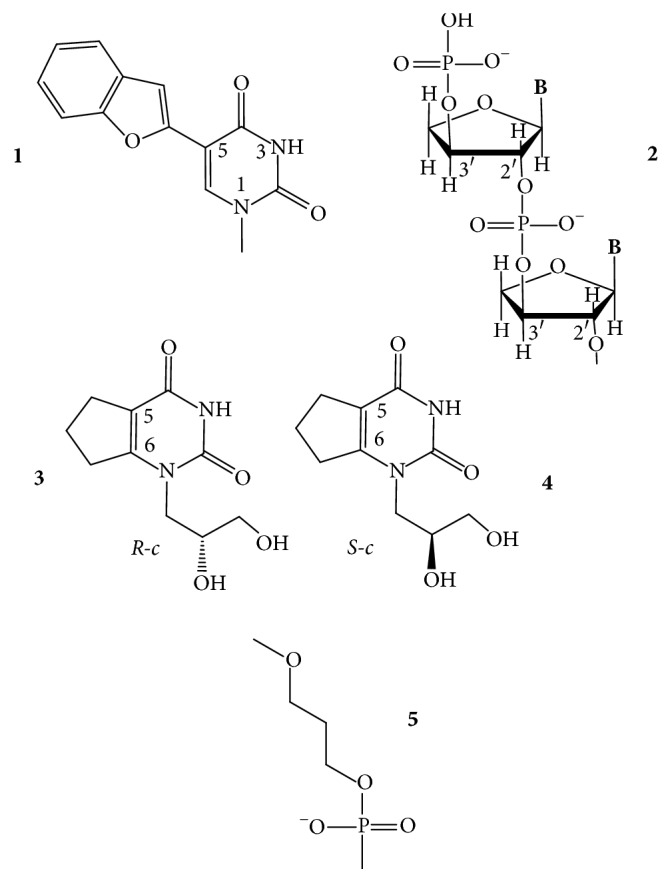
Structures of 5-(benzofuran-2-yl)uracil (**1**); threose dinucleotide (**2**); R- (**3**) and S-isomer (**4**) of acyclic-uracil derivatives and the n-propyl spacer (**5**).

*5-(Benzofuran-2-yl)uracil*. Tanpure and Srivatsan [53] reported that the 5-(benzofuran-2-yl)uracil (Figure 5,** 1**) can be used to monitor the formation of GQs from htel DNA and RNA sequences. The uracil probe exhibited 4- to 9-fold fluorescence intensity enhancements in the GQ compared with its duplex structure. The effect depended on the GQ topology and on the position of the modification. The probe minimally perturbed the GQ topology and stability and could distinguish the antiparallel, hybrid, and parallel topologies of DNA and RNA GQs from the corresponding duplexes. UV thermal melting profiles showed that the unmodified and the modified GQs had similar *T*_*m*_ values, within ≤2°C, even in different ionic conditions.

*Threose Nucleotide (TNA)*. The sugar analog *α*-L-threofuranosyl nucleotide (TNA for threose nucleic acid) (Figure 5,** 2**) has been examined if the oligonucleotides containing this shorter internucleotide building unit can also form GQs [54]. Despite the modified backbone repeat unit, which is one atom shorter than that found in DNA and RNA, TNA could self-assemble into stable tetramolecular GQ structures that are similar in thermal stability to the 2′-deoxyribose-containing [TG_4_T]_4_. Unlike DNA, the TNA GQs formed equally well in either Na^+^ or K^+^ ions [54].

*Acyclic-Uracil Derivatives*. The R-stereoisomer of an acyclic uracil containing a cyclopentane ring fused at positions 5 and 6 of the uracil ring (R-c, Figure 5,** 3**) had been introduced by Borbone et al. [55] into position T7 or T12 (TGT and the TT loops) of the TBA oligodeoxynucleotide. The resulting two aptamers folded into the typical antiparallel chair-like GQ structures formed also by the native TBA in K^+^ buffer (Figure 2). The acyclic residue increased the thermal stability of the resulting GQs with respect to the TBA GQ, by 1°C and 4°C, respectively. Anticoagulant activity of the TBA-T7 was more potent than the TBA's in prolonging clotting time [55]. Later, the research group incorporated both the S and the R stereoisomers (S-c and R-c; Figure 5,** 3**,** 4**) into the same TBA sequence into positions 3, 7, and 12. Positions 3 and 12 are located in the two TT loops, and position 7 is located in the TGT loop. At these locations, both isomers of the acyclic-T derivative enhanced the thermal stability of the resulting GQs and all adopted the same antiparallel topology as the parent TBA GQ did. *T*_*m*_ of the unmodified GQ, 50°C, was raised to 54-55°C by the R-derivatives and to 51–56°C by the S-derivatives [56] (it is not known how these derivatives would influence the thermal stability in the other three T positions of the loops).

*n-Propyl Spacer*. A three-carbon spacer (Figure 5,** 5**) replaced the base-sugar residues of T3, T7, and T12 loop positions of the TBA GQ. The flexible abasic sites increased *T*_*m*_ of the unmodified TBA GQ (49°C) by 6°C at each of the three positions and did not alter the antiparallel chair folding topology of the unmodified GQ. The modifications improved the thrombin clotting time [57].

*Other Stabilizing Analogs*. A novel fluorescent TBA analog has been created by conjugation with a dansyl probe at the 3′-end and a *β*-cyclodextrin residue at the 5′-end. The bis-conjugated TBA analog could fold into the chair-like, antiparallel GQ structure in K^+^, typical of the native TBA GQ, and retained its thrombin-binding properties as well. The terminal appendages only marginally affected the conformational features of the TBA GQ. Folding into GQ was associated with a net fluorescence enhancement due to encapsulation of the 3′-dansyl group into the apolar cavity of the *β*-cyclodextrin at the 5′-end. This novel analog of the TBA GQ demonstrated its potential as a diagnostic tool for thrombin recognition and also provided a useful basis for the design of suitable aptamer-based devices for theranostic applications, that is, allowing simultaneous detection and inhibition or modulation of the thrombin activity. The terminal double conjugation increased *T*_*m*_ of the native TBA by about 10°C [58].

The short TG_4_T oligodeoxynucleotide forms a stable tetramolecular parallel-stranded GQ in K^+^ solution. The 3′- and/or the 5′-ends of four TG_4_T molecules have been linked together by a non-nucleotide-type, tetra-end-linker (TEL) by Oliviero et al. [59]. The TEL-[TG_4_T]_4_ architectures were able to form parallel GQs regardless of the TEL size and the structural orientation of the oligonucleotide branches. The TEL-[TG_4_T]_4_ structures that had longer TEL were more thermostable than those with the shorter TEL.

A perylene group, a five-membered fused aromatic structure, has been linked to the nonbonded oxygen of the phosphodiester linkage between dG-dG, dG-T, and T-T dinucleotides, and a single perylene-modified dimer was introduced in the TBA and the 20-mer (TGGGT)_4_ oligonucleotides to develop fluorescence anisotropy GQ probes. Single modifications showed little effect on GQ conformation and the thermostability increased in both K^+^ and Pb^++^ if the perylene moiety was not located between the dG stacks. With some modified sequences, *T*_*m*_ of the resulting GQ increased by 32°C. In most cases, the GQs were more stable in Pb^++^ than in K^+^ with *T*_*m*_ increasing up to 26°C. The large stabilization was probably due to the interaction of perylene with the G-quartet plane and a more compact structure was induced by Pb^++^. CD spectra of the (TGGGT)_4_ GQ and the modified analogs showed parallel topology in the presence of K^+^ with a positive peak near 265 nm and a negative one near 240 nm. The TBA and its analogs displayed positive peaks near 245 and 295 nm and a negative one near 265 nm, typical of the antiparallel GQ structures. In the presence of Pb^++^, the positive peaks of 265 nm and 295 nm of the structures were red-shifted, indicating the Pb^++^-stabilized antiparallel GQs in both scaffolds [60].

Non-nucleoside-type linkers, such as propanediol, octanediol, or hexaethylene-glycol, that replaced the whole TTA loops connecting the four GGG tracts of an htel GQ induced the formation of parallel GQs, like the single-nucleotide loops do, as Risitano and Fox have reported [61]. A special feature of these loop-replaced structures was that *T*_*m*_ of all of these was above 37°C in the presence of 10 mM Li^+^ alone, without added K^+^, in contrast to any wild-type htel GQ. In 40 mM K^+^, all loop-replaced htel analogs melted at higher temperatures than the wild-type TTA loop-containing 5′F-G_3_TTAG_3_TTAG_3_TTAG_3_-Q GQ did (*T*_*m*_ 69°C), where F and Q stand for the fluorophore and the quencher molecules, respectively.

## 3. Stabilization or Destabilization of a GQ by the Same Synthetic Nucleotide Analog

Depending on the folding topology, GQs contain nucleosides of* anti* and* syn* N-glycosidic linkages, which connect the base and sugar moieties. In most cases, the parallel GQs of any molecularity contain all* anti* nucleosides, and the antiparallel and hybrid architectures (Figure 2) comprise both types of natural nucleosides. Although the thermodynamic variance between the two conformation states is small, in the range of 1-2 kcal/mol, forcing a nucleoside from* syn* to* anti* or vice versa does affect the thermodynamic stability of a GQ and generally leads to destabilization. In some cases, it also induces topological rearrangement of the GQ. In this way, all 8-substituted 2′-deoxyguanosines whose thermodynamically preferred glycosidic conformation is* syn*, such as the 8-bromo-dG and 8-methyl-dG, if incorporated into* syn* dG positions generally enhance the thermodynamic stability of the native GQ, whereas if incorporated into* anti *dG positions they generally reduce the stability. Similarly, if the* anti* stabilized sugar analogs substitute for* syn *dG positions, these not only destabilize the structure but can also alter the folding topology. For instance, an antiparallel fold, such as that of the monomolecular TBA GQ, transforms into a nonmonomolecular parallel fold on the effect of such substitutions. Another factor in the stabilization-destabilization is the sequence position within the potential GQ-forming oligonucleotide. The same nucleotide analog, independently of the* syn*/*anti* isomerism, can increase the stability at a given position and decrease it in another location. This alternate effect depends on the role of the substituted position in the stabilization of the folded native structure.

### 3.1. Incorporation of Base Derivatives 

#### 3.1.1. Purine Analogs

**Figure 6:**
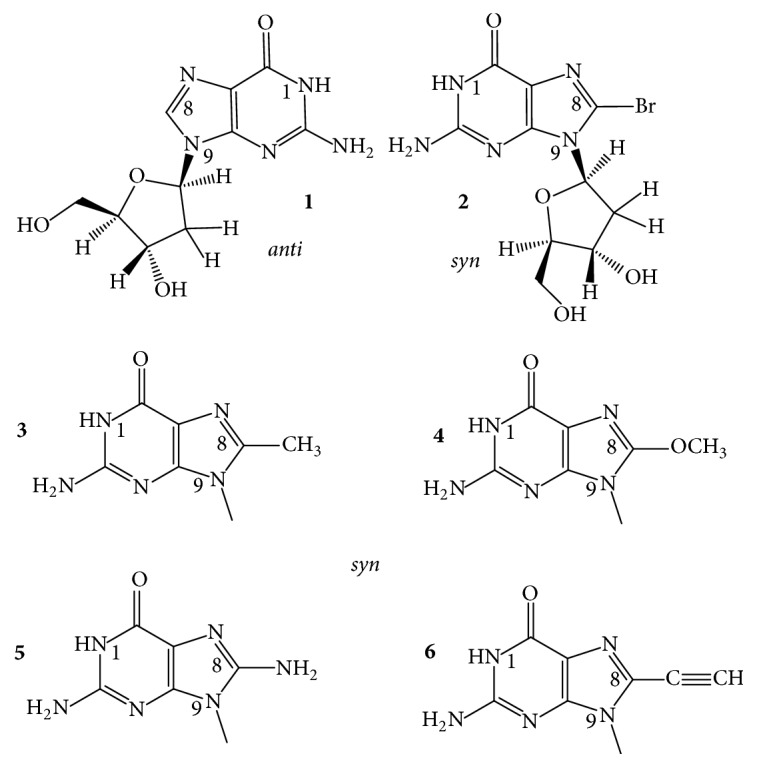
Structures of* anti* 2′-deoxyguanosine (**1**);* syn* 8-bromo-dG (**2**); 8-methylguanine (**3**); 8-O-methylguanine (**4**); 8-aminoguanine (**5**); 8-propynylguanine (**6**).

In GQs, the position C8 and also one hydrogen atom of NH_2_ of C2 of guanine are the only suitable sites for modification that may not cause major deformation of the folding topology. Substituents of these positions point towards the grooves, towards the solvent, away from the Hoogsteen-type circular double H-bonding structure, and away from the central cavity of the stacked G-tetrads. C8 of guanine has been the most frequently modified position. The energetically preferred N-glycosidic torsion angle of 2′-deoxyguanosine (dG) is in the* anti* range (Figure 6,** 1**), whereas bulky atoms, like the halogens, the oxygen, and groups that are large enough to sterically interfere with the 5′-OH of the sugar moiety, turn the glycosidic bond into the* syn*-region (Figure 6,** 2**–**6**) [62–64]. The difference between these conformations has been estimated to be ~1-2 kcal/mol [33]. When the 8-substituted-dG replaces an originally* syn *dG, it can generally stabilize the scaffold for which the best example is 8-bromo-dG.

*8-Bromo-*2′*-deoxyguanosine (b*r^8^*dG)*. Dias et al. [64] described in 1994 that incorporation of br^8^dG into the 20-mer (T_3_G_2_)_4_, which folded into a monomolecular antiparallel chair-type GQ (Figure 2,** 2**), stabilized the (T_3_G_2_)_4_ by 5-6°C in the *T*_*m*_ value when* syn* dGs were switched to br^8^dGs. Free energy difference (ΔΔ*G*°_25_) between the parent and the* syn *br^8^dG-modified GQs was −1 kcal/mol. The br^8^dG-induced destabilization also came first from this study. The (T_3_G_2_)_4_ GQ was destabilized by 3–6°C in its *T*_*m*_ when the* anti* dG nucleotides were replaced by br^8^dG. Free energy difference between the parent and the eight* syn-* and* anti* substituted br^8^dG-modified GQs was +1 or −1 kcal/mol. Xu et al. [65] found in 2006 that br^8^dG stabilized the GQ fold of the 22-mer human telomere sequence A(htel-21) or AG_3_(TTAG_3_)_3_ in K^+^ solution when br^8^dG was incorporated in place of a* syn *dG position of the 3-tetrad monomolecular GQ. The enhanced thermodynamic stability was supposed to be the driving force for the originally polymorphic folds [8] (Figure 2,** 1**–**4**) to convert into a more stable single form. As a result, br^8^dG has since been frequently used to stabilize particular DNA and RNA GQ conformations for structural studies carried out with CD, UV, NMR spectroscopy, and other techniques [66–93]. In certain cases, br^8^dG could stabilize a GQ also in* anti* G positions. Aviñó et al. [94] substituted the TBA GQ (Figure 2,** 7**) with br^8^G, which stabilized the GQ when substituted for the* syn* position G5, by 7.3°C, and interestingly also by the double substitutions for G2, G11, by 3.6°C, which were both* anti* positions. The TBA GQ remained antiparallel. The analog destabilized the GQ in the* anti* position G2 by 2.5°C and also by double substitutions of the* anti* positions G2 and G6 by 8.8°C [94].

A remarkable example of using br^8^dG for structure studies was the stabilization of individual topologies in telomeric GQs. Attaching flanking nucleotides to the basic htel-21 DNA sequence of G_3_(TTAG_3_)_3_ induces diverse folds [8]. For instance, TA(htel-21) was found by NMR technique to mostly fold into the hybrid-1 form (Figure 2,** 3**), whereas the TA(htel-21)TT adopted the hybrid-2 topology (Figure 2,** 4**) as the dominant, but not the only form. The hybrid structures are generally polymorphic in solution and are interconvertible due to the small energetic differences between them [84]. Incorporation of br^8^dG into selected* syn *dG position(s) converted the polymorphic fold into a stabilized majority fold. For example, the* syn *dG16 in the middle tetrad of the TA(htel-21) and the* syn *dG15 in one of the terminal tetrads of the TA(htel-21)TT GQs greatly stabilized their respective major topologies, according to Phan et al. [71]. Another noteworthy illustration for the effect of br^8^dG was the induction of conformational changes in GQs. Using the same 25-mer TA(htel-21)TT, An et al. [87] incorporated two br^8^dGs into the sequence. The dual incorporation induced exclusively either the hybrid-1 or the hybrid-2 fold in K^+^ (wt for the wild-type):

  Wild-type (wt): TA GGG TTAGGG TTAGGG TTAGGG TT
  br^8^dG in G16, G21: TA GGG TTAGGG TTAG$\underline{br^{8}G}$G TTA$\underline{br^{8}G}$GG TT hybrid-1
  br^8^dG in G10, G15: TA GGG TTAG$\underline{br^{8}G}$G TTA$\underline{br^{8}G}$GG TTAGGG TT hybrid-2

br^8^dG impacted both the topology and stability when incorporated into tetramolecular parallel GQs (Figure 2,** 6**). TG_3_T molecules in K^+^ fold into [TGGGT]_4_. Esposito et al. [67] separately substituted each G of TG_3_T with br^8^G. All three modified sequences assembled into tetramolecular GQs; the stabilities and topologies were, however, different. CD spectrum of [T$\underline{br^{8}G}$GGT]_4_ showed, surprisingly, antiparallel characteristics and its thermal stability was much higher, by 13.3°C, than that of the native parallel tetramolecular scaffold. The [TG$\underline{br^{8}G}$GT]_4_ formed parallel GQ architecture, as the native sequence did, and stabilized the native structure by 3.6°C. The TGG$\underline{br^{8}G}$T proved unstructured, and no *T*_*m*_ could be determined. It was also an unusual finding that the br^8^G tetrads were all* syn* with each tetramolecular analog, while the other two G-tetrads of the GQ were* anti*, as in the case of the unmodified [TGGGT]_4_.

*8-Methyl-*2′*-deoxyguanosine (m*^8^*dG)*. Another stabilizing guanine analog is the 8-methylguanine nucleoside (m^8^dG) (Figure 6,** 3**), and as with the br^8^dG, it is stabilizing only if it is substituted for* syn *dGs. m^8^G has been, however, much less utilized than the br^8^G. He et al. [33] incorporated a methyl (and a 1-propynyl) group into the C8 positions of G1, G5, G10, and G14 of the 15-mer TBA DNA oligonucleotide (Figure 2,** 7**), where positions were all* syn*. Thermal stability of the unmodified TBA GQs, 55°C, was increased to 70°C by these substitutions. Concomitantly, the substitutions increased the thrombin-binding activity as well, presumably by the stabilization of the chair-like structure. Xu and Sugiyama [95] incorporated the m^8^dG into the 18-mer CGGGGGGTTTTGGGCGGC of the G-rich termini of retinoblastoma gene DNA. Thermal stability of the two-tetrad antiparallel GQ increased when* syn *dGs were replaced and decreased when an* anti *dG was substituted by m^8^dG. The GQ stability pays energy penalty for accommodating a* syn *dG analog in place of an* anti* dG. Later, m^8^dG was incorporated into the tetramolecular GQ-forming TG_3_T and TG_4_T oligodeoxynucleotides (Figure 2,** 6**). Using [TGGGT]_4_, Virgilio et al. [96, 97] and Tran et al. [98] determined that m^8^dG had a position-dependent effect on folding arrangements. N-glycosidic linkages of the G and T nucleosides are all* anti* in the native fourfold parallel GQs. In position 2, however, m^8^dG formed an all-*syn* tetrad that resulted in an antiparallel-type CD spectrum in K^+^, that is, a large positive band near 290 nm and a negative one near 260 nm (Figure 3). The m^8^dG-tetrad in position 3 was all* anti*, the GQ was parallel, and the CD spectrum showed large positive peak at 260 nm and a small negative one around 240 nm. m^8^dG in position 4 hindered the GQ formation. Results were similar to those observed with br^8^dG. The authors also studied m^8^dG in position 2 of the anti-HIV aptamer TGGGAG (Hotoda aptamer) and its 3′ T-extended sequence TGGGAGT and observed thermal stabilization. Again, all-*syn* m^8^dG tetrads were observed in both GQs; however, the topologies remained parallel [99]. Structure and kinetics of formation of the tetramolecular GQ model were investigated using m^8^G in another study. m^8^dG at the 5′-end of the short oligonucleotide accelerated GQ formation by 15-fold relative to the unmodified oligonucleotide [98]. In a recent study, Zhao et al. [100] substituted dGs by m^8^dGs in the GQs formed by an htel, the TBA, and the c-myc promoter's G-rich pu27 sequences. m^8^dG proved significantly stabilizing in those cases when it replaced* syn* dGs and the stabilization was cumulative when two or three m^8^dGs were substituted for* syn* dGs. However, m^8^dG proved destabilizing when an* anti* dG was replaced.

*8-O-Methyl-*2′*-deoxyguanosine (om*^8^*dG)*. Lech and coworkers [80] studied the influence of 8-O-methylguanine deoxyguanosine (Figure 6,** 4**) on GQ structures using the 24-mer model TT(htel-21)A that forms a predominant hybrid-1 topology (Figure 2,** 3**) in K^+^ solution [101]. Inserted one by one into six different positions (bold) into the 24-mer TT**G**GGTTA**G**GGTTA**GG**GTTA**GG**GA, each analog elevated the thermal stability of the GQ by an average of 3.1 ± 1.4°C, with slight positional effects. Except for position 22, each site was* syn*. The guanine analog in the* anti *dG22 position also elevated the stability, although somewhat less than in the** 21*** syn* position. The hybrid-1 topology was not basically changed by om^8^dG.

*8-Amino-*2′*-deoxyguanosine (n*^8^*dG)*. The effect of the* syn*-preferring n^8^dG on GQ stability (Figure 6,** 5**) was first described using the TBA GQ by De La Osa et al. [102]. The analog was placed (for MD calculation) and incorporated into position 2, which is an* anti* dG position of the GQ. Molecular dynamics and thermodynamic integration calculations (MD/TI) suggested that the n^8^dG substitution led to destabilization by 1.2–1.9 kcal/mol in the ΔΔ*G*° value, and the nature of the central cation (Na^+^ or K^+^) did not have substantial effect. CD-based melting profiles confirmed the MD/TI calculations. The unmodified GQ had a *T*_*m*_ value of 46°C, while the n^8^dG-modified one had 39°C. The loss in folding free energy by the single substitution corresponded to 1.2 kcal/mol, in perfect agreement with the MD/TI calculations. This destabilization was enthalpic in nature, with a ΔΔ*H*° of 5.5 kcal/mol. n^8^dG was later incorporated into the tetramolecular parallel GQ models built from TG_4_T and TG_5_T and an unusual position-dependent effect was observed by Gros et al. [73]. In the [TG_n_T]_4_, the n^8^dG tetrads reduced the thermostability in three of the four G positions and elevated the stability only in position 3 of the TG_4_T sequence. With another, on the 5′-end covalently linked tetramolecular assembly, Ferreira and coworkers [103] found that inserting a single n^8^G only into one strand in position 2 or 3 stabilized the parallel GQ. In both cases, n^8^G-G-G-G tetrads were formed:

  3′-T$\underline{n^{8}G}$GGGTT-5′-TB-[5′-TTGGGGT-3′]_3_,
  3′-TG$\underline{n^{8}G}$GGTT-5′-TB-[5′-TTGGGGT-3′]_3_,

where TB was the Trebler linker [O-phosphate-CH_2_-C(CH_2_OCH_2_CH_2_CH_2_O-phosphate)_3_]. With the intramolecular, mostly hybrid-1 GQ of TT(htel-21)A, Lech et al. [80] found that n^8^dG was only marginally stabilizing when incorporated into the* syn *dG positions of 3, 9, 15, 16, and 21 with the average Δ*T*_*m*_ of 1.1 ± 1.1°C. According to the CD spectra the hybrid-1 type, topology (Figure 2,** 3**) apparently was not changed by any of these substitutions.

*8-Propynyl Derivatives of *2′*-Deoxyguanosine*. The triple C-C bond-containing propynyl group in conjugation with heteroaromatic bases, such as the uracil in duplexes [104] as well as in the TBA GQs [52], greatly enhanced the stability of the macromolecular structure through strengthening the stacking. The propynyl group in conjugation with guanine (Figure 6,** 6**) also stabilized the GQ, which was again the TBA GQ, when incorporated into all four* syn* dG positions, G1, G5, G10, and G14, of the 15-mer sequence. The substitutions increased the thrombin-binding activity of modified TBA GQ, as compared to the wild type, probably due to the stabilization of the intramolecular GQ structure. Larger substituent groups in these positions, like phenyl-ethynyl, however, decreased the activity, probably due to steric hindrance [33].

**Figure 7:**
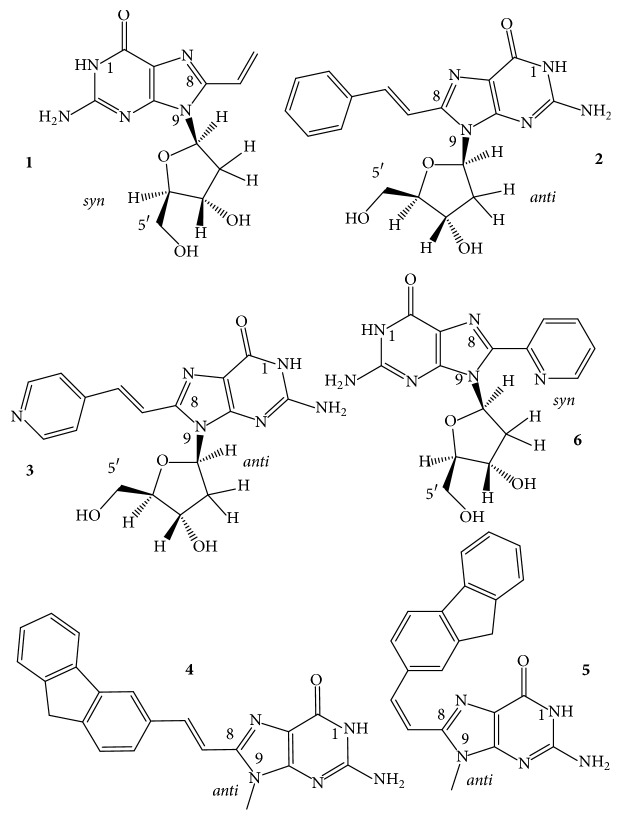
Structure of the* syn* 8-vinyl-2-deoxyguanosine (**1**); the* anti* 8-(2-phenylethenyl)- (**2**) and 8-[2-(pyrid-4-yl)-ethenyl]-dGs (**3**); the* cis* (**4**) and* trans* isomers (**5**) of the* anti* 8-vinyl-substituted-dGs; and the* syn *8-(2-pyridyl)-dG (**6**).

**Figure 8:**
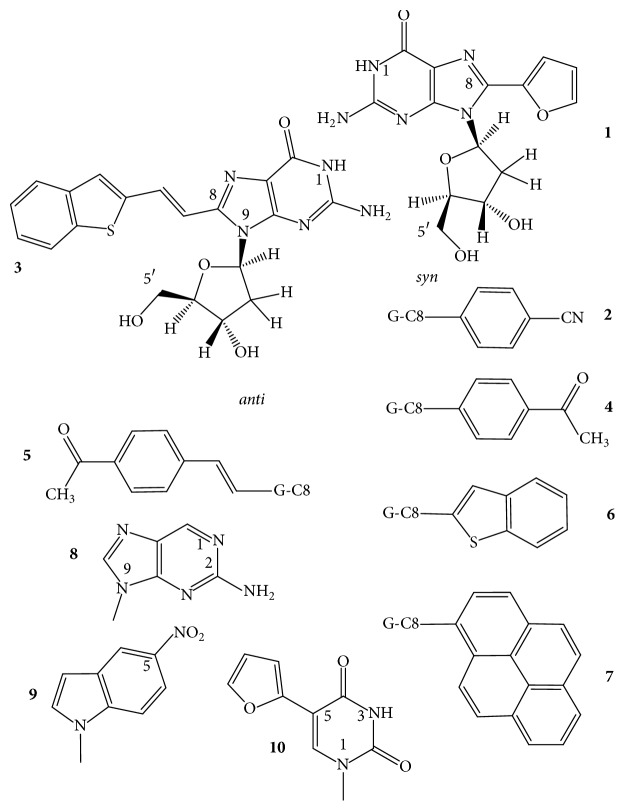
Structures of* syn* 8-furyl-dG (**1**); 8-(4-cyanophenyl)guanine (**2**);* anti* 8-vinyl-benzothienyl-dG (**3**); 8-acetylphenylguanine (**4**); 8-vinyl-acetylphenylguanine (**5**); 8-benzothienylguanine (**6**); 8-(pyren-1-yl)guanine (**7**); 2-aminopurine (**8**); 5-nitroindole (**9**); and 5-furyl-2′-deoxyuridine (**10**).

*8-Vinyl-*2′*-deoxyguanosine (v*^8^*dG) and Its Derivatives*. The fluorescent base 8-vinylguanine (v^8^G) (Figure 7,** 1**) proved to be a good alternative to the widely used fluorescent marker 2-aminopurine (n^2^Pu, Figure 8,** 8**) [105, 106]. Nadler et al. [105] incorporated the v^8^dG into positions G3, G15 (middle tetrad), and G4 (terminal tetrad) of the htel-23 A(htel-21)T. In 135 mM Na^+^ solution, the two middle tetrad substitutions elevated the thermal stability of the wild type (*T*_*m*_ = 56°C) by 3-4°C and v^8^G in one of the two terminal tetrads by 2°C. In 100 mM K^+^ only the vinyl analog of the middle tetrad (position 15) increased the stability by 5°C of the unmodified GQ (*T*_*m*_: 61°C). CD spectra of the three modified GQs in Na^+^ looked hybrid types contrary to the wild-type's antiparallel spectrum (Figure 3). The wild-type htel-23 folds into hybrid conformations in K^+^ solution. The middle tetrad-modified GQ studied followed the trend in K^+^, whereas the CD spectrum of the terminal tetrad-modified GQ was antiparallel type [105]. 8-Vinylguanine was also attached to 2-aminoethylglycine, creating a peptide nucleic acid (PNA) building block. This allowed the differentiation between topologies of GQs based on fluorescence intensity. v^8^dG was found to be capable of adopting both* syn* and* anti *conformations required by distinct GQ structures [106].

Aromatic derivatives of 8-vinylguanine, the 8-(2-phenylethenyl)- and 8-[2-(pyrid-4-yl)-ethenyl]guanines (Figure 7,** 2**,** 3**), have been studied by Dumas and Luedtke [107] by incorporating them into positions 9 and 23 of the 24-mer TT(htel-21)A. Although v^8^dG can adopt both* syn* and* anti* conformations [105, 106], the two aromatic derivatives of v^8^dG adopted only* anti* glycosidic conformation in solutions, like dG does, contrary to other 8-modified dGs, such as br^8^-, m^8^-, n^8^-, or o^8^dG. Due to the conjugated vinyl tethers, the bulky aryl and heteroaryl groups are detached far enough from dG not to hinder the energetically preferred* anti *orientation of dG. The GQ of TT(htel-21)A has been described to fold into hybrid-1 topology (Figure 2,** 3**) in K^+^ and into the antiparallel topology in Na^+^ (Figure 2,** 1**) [107]. The position 9-substituted 24-mer GQ was stabilized by the two ethenyl derivatives by 7-8°C in Na^+^ and 5–8°C in K^+^; however, in position 23, the GQs were destabilized by 1–6°C in Na^+^ and 6–8°C in K^+^. In position 9, the two G-analogs did not change the CD spectrum of the wild-type GQs (hybrid), whereas the position 23-substituted GQs showed antiparallel-type spectra in K^+^. In Na^+^, the modified GQs remained antiparallel like the wild-type htel-24 GQ did [107]. 8-Fluoroenylvinylguanine (Fv^8^G), another derivative of v^8^G, was also incorporated into GQ-forming oligonucleotides. Ogasawara and Maeda [108] developed a light-controlled reversible folding-unfolding GQ structure with this analog based on the* cis*-*trans* isomerization of a photochromic nucleobase Fv^8^G (Figure 7,** 4**,** 5**), incorporated into the 15-mer TBA sequences, the GGTTG$\underline{Fv^{8}G}$TGTG$\underline{Fv^{8}G}$ TTGG and G$\underline{Fv^{8}G}$ TTGGTGTGGTTG$\underline{Fv^{8}G}$.

The native TBA sequence folds into a chair-type, two-tetrad antiparallel GQ in K^+^ (Figure 2), and its CD spectrum was only slightly changed by the* trans*-Fv^8^G (Figure 7,** 4**) in* anti *dG positions, G6 and G11 and G2 and G15. *T*_*m*_ of the modified GQs increased by ~10°C, up to ~60°C. On irradiation at 410 nm, the* trans*-Fv^8^G changed into the* cis* form (Figure 7,** 5**), which resulted in unfolding of the stable GQs. Irradiation at 310 nm reversed the unfolding. 8-(2-Pyridyl)guanine (Py^8^G, Figure 7,** 6**) is a highly fluorescent compound that has been used to study the folding of the 24-mer TTG_3_(TTAG_3_)_3_A GQs [109]. Positions G9 and G23 of terminal tetrads were* syn* in Na^+^; still the* syn *Py^8^dG affected the thermostability in altered ways: 9-Py^8^dG increased *T*_*m*_ of the wild-type GQ by 7 and 10°C in Na^+^ and K^+^, respectively, whereas 23-Py^8^dG reduced the wild-type's *T*_*m*_ by 2 and 9°C, respectively. Apparently, the glycosidic orientation of the Py^8^dG was not related to these effects. 9-Py^8^G in K^+^ did not change the wild-type's hybrid fold; however, 23-Py^8^G induced a conversion into antiparallel topology in K^+^.

The “push-pull” emissive fluorophores, which can exhibit environmentally sensitive quantum yields due to excited-state proton-transfer reactions with bulk solvent, were thoroughly investigated by Manderville's and Wetmore's groups. 8-Furyl- and 8-(4-cyanophenyl)-2′-deoxyguanosines (Figure 8,** 1**,** 2**) were used to monitor the duplex-GQ exchanges. In a* syn *dG position of the TBA GQ (pos. 5), the* syn* 8-aryl dGs significantly stabilized the GQ in both Na^+^ and K^+^ solutions, whereas they substantially destabilized it when incorporated into an* anti *dG location (pos. 6) [110, 111]. The 8-furyl- and 8-vinyl-benzo(b)thienyl-dGs (Figure 8,** 1**,** 3**), which, based on analogy, are assumed to be* syn* and* anti* dGs, respectively, were incorporated into both* syn* and* anti *dG positions of the TBA DNA. 8-Furyl-dG in the* syn* positions of 10 and 14 elevated *T*_*m*_ of TBA GQ, while the more lipophilic benzothienyl derivative decreased it slightly in the* syn *G5 position and more extensively in the* anti *G6 position. Double labeling with furyl and benzothienyl derivatives enhanced the destabilization when one of the analogs was in* anti *position; however, when both probes substituted for* syn *dG positions, the wild type was significantly stabilized [112]. The push-pull phenomenon in the TBA GQ was also studied with acetylphenyl, benzo[b]thienyl, quinolyl, pyren-1-yl (Figure 8,** 7**), and the 8-vinyl tethered derivatives of acetylphenyl and benzo[b]thienyl attached to C8 of dG (Figure 8). The dG analogs were substituted for* syn* dGs of the TBA DNA sequence. The aromatic and heteroaromatic groups with the aryl ring attached directly to C8 of guanine thermodynamically favor the* syn* glycosidic conformations and when these analogs substituted for the* syn* dG5 they increased the thermostability of the wild-type TBA GQ. The two aromatic analogs tethered to guanine base via the conjugated vinyl group favored* anti* glycosidic torsion angle (Figure 8,** 3**,** 5**). The* anti *8-vinyl-acetylphenyl-dG slightly decreased *T*_*m*_ of the wild type in the* syn *dG5 position but moderately elevated it in the* anti *dG6 position [113]. Similarly, the fluorescent 8-pyrrolyl-, 8-furyl-, 8-thienyl-, 8-benzofuryl-, 8-indolyl-, and 8-benzothienyl-dGs were also incorporated into the TBA sequence in another study. These nucleosides also preferentially adopt* syn* conformation in solution and their insertion into the* syn *dG5 enhanced the thermal stability of the parent GQ in K^+^ solution by 1–11°C and did not perturb the thrombin-binding affinity. 8-Thienyl-dG was found to increase the thermostability in each of the four* syn* positions by 7–10°C and its double incorporation by 18.5°C, and substituting all four* syn *dGs, Δ*T*_*m*_ was higher than 36°C [114]. Stability changes resulting from the* syn *versus* anti* glycosidic conformations of dG derivatives were similar to 8-(p-cyanophenyl)-dG in TBA and 8-furyl-dG in A(htel-21) GQ systems [115]. 8-Furylguanine was also incorporated into each of the three tetrads of A(htel-21) and the effects of salts and cosolvents (acetonitrile, PEG-600, and N-methyl-mesoporphyrin) were examined on the structure. In addition to the influence of the* syn *and* anti* positions on stability, the position of the tetrad substituted affected the stability too [116]. The effect of 8-vinyl-acetylphenyl- and 8-vinyl-benzothienyl-dGs on the TBA GQ system was also investigated for the detection of divalent metal ions. Based on its emission sensitivity to Pb^2+^, the vinyl-benzothienyl derivative proved to be an effective and selective sensor for Pb^2+^, having higher affinity for Pb^2+^ than for Na^+^, K^+^, or other bivalent cations of biological importance [117].

The C8-aryl- and heteroaryl substituents, such as C8-phenyl, pyridine, thiophene, and furan of dG, were also studied by Dumas and Luedtke [118], who used the changes in fluorescence to characterize the metal-binding affinity and specificity of the 8-substituted guanines in duplex and GQ DNAs as well as to study the effect on stability. Thermal stability of the GQ formed by the cKit sequence AG_3_AG_3_C**X**CTG_2_**X**AG_2_AG_3_ substituted in positions 10 and 15 (X) was investigated. 8-(2-Pyridinyl)guanine (Figure 7,** 6**) decreased *T*_*m*_ of unmodified GQ (64°C) by 2–4°C in positions 10 and 15. This analog has also been used to study the folding properties of the 24-mer TT(htel-21)A GQ [109]. Position G23 in the 3′-terminal tetrad was* syn* in Na^+^; still the* syn*-Py^8^dG23 reduced the wild-type's *T*_*m*_ by 2 and 9°C in Na^+^ and K^+^, respectively. Apparently, the glycosidic orientation of the Py^8^dG was not related to the effect. The Py^8^G23 induced a conversion of the hybrid fold(s) into antiparallel topology in K^+^.

*2-Aminopurine (n* ^2^ *Pu)*. The fluorescent 2-aminopurine (n^2^Pu, a G or A analog) (Figure 8,** 8**) incorporated into the loop sequence of a potentially GQ-forming oligodeoxynucleotide has significantly enhanced fluorescent emission upon the formation of the GQ. (Its fluorescence is quenched in duplex DNA due to the stacking with flanking bases and becomes comparable to that of the free n^2^Pu upon the formation of GQ as the *π*-stacking within the loops is distorted and effective quenching cannot occur.) n^2^Pu has been widely used in structural analysis of GQs. For example, for conformational studies, n^2^Pu was substituted separately for all four adenines in A(htel-21) [84, 119–121]. The analog did not change the folding and only slightly reduced the thermal stability of the parent GQ [122], while significant changes were observed in fluorescence intensity of n^2^Pu depending on whether it was present in the GQ or in the duplex formed with the complementary strand. n^2^Pu could also distinguish between the basket-type and propeller-type GQs. 2-Aminopurine has also been used to develop a sensitive fluorescent GQ assay for uracil DNA-glycosylase activity [123]. 2-Aminopurine was also substituted for Ts of the loops the 15-mer TBA and the (G_3_T)_3_G_3_ oligonucleotides to develop fluorescent detection methods. The two oligomers folded into antiparallel and parallel GQs, respectively, in K^+^. The substitutions did not change the original topologies but changed the stabilities of the wild-type Qs. *T*_*m*_ of 48°C for wild-type TBA GQ in 50 mM KCl was increased by 2°C when substituting for T3 and T2 of the loops, in which positions the T is stacked to the core [18–20]. In the other T positions, the n^2^Pu reduced the stability to around 40°C. *T*_*m*_ of the (G_3_T)_3_G_3_ GQ measured in 10 mM KCl was 90°C and was reduced in all three loop T positions by n^2^Pu. Single substitutions caused 4-5°C decrease in *T*_*m*_, while double and triple substitutions caused a decrease of 12°C and 16°C, respectively [124]. Similarly, n^2^Pu substituting for G8 of the central TGT loop just slightly reduced the *T*_*m*_ value of 52.4°C of the native GQ to 51.6°C, but the modified TBA GQ proved more stable based on the free energy change (Δ*G*°_20_) value from −2.3 to −3.6 kcal/mol [125].

*5-Nitroindole*. Tsvetkov et al. [126] incorporated the universal base 5-nitroindole (Figure 8,** 9**) into various positions of the TBA oligonucleotide. All modified TBAs formed antiparallel GQ structures and retained the ability to inhibit thrombin. UV absorption-based thermal stability of T7 substitution resulted in *T*_*m*_ of 43.0°C and of the T9 in 38.5°C, while *T*_*m*_ of the native TBA GQ was 51.9°C. On the other hand, replacement by 5-nitroindole of the* anti* G8 retained the stability of the native TBA GQ and this substitution substantially increased the clotting time and resulted in a twofold lower IC_50_ value, as compared to the unmodified TBA GQ. Attachment of 5-nitroindole either to the 5′ or to the 3′-terminus did not change the stability of the unmodified GQ, 51.1 and 51.2°C, respectively [126].

#### 3.1.2. Pyrimidine Derivatives

Loop length and composition are the major factors that determine the stability and folding features of natural GQs [61, 127, 128]. For instance, a single one-nucleotide loop generally keeps the GQ in parallel topology [129, 130]. With increasing loop lengths, thermodynamic stability generally decreases [128]. Effect of loop substitutions on GQ structures has been widely studied, but the majority of the investigations focused on the mutation by natural nucleotides, T/U, C, A, and I, to increase the loop length and change the sequence. The TBA DNA was among the most mutated GQs due to its stable and straightforward conformation [10]. In addition to 2-aminopurine, examined in the preceding section, those few loop base modifications are discussed here that have been carried out using the synthetic nucleotide analogs only, which increase or decrease the stability depending on the position of substitution. The natural base lesions that also form in the loops of GQs, such as o^8^A, hm^5^U, and AP sites, are reviewed in Section 5.

The TBA DNA's six loop thymidines were replaced by a fluorescent furyl derivative, the 5-furyl-2′-deoxyuridine (Figure 8,** 10**), to demonstrate the analog's ability to determine the positional impact of each thymine on the stability and thrombin-binding activity of the TBA GQ [131]. Earlier NMR studies suggested that T4 and T13 stack strongly with the neighboring G-tetrad and T9 of GGT**T**GGTG**T**GGT**T**GG interacts with G8 of the TGT loop and with the G-tetrad. The T3, T7, and T12 do not interact with adjacent nucleotides and point outwards, towards the solvent [18–20]. Thermal stability of the modified TBA GQs showed good correlation with the steric predictions of the NMR studies. In K^+^ solution, the furyl analog in positions 4, 9, and 13 enhanced *T*_*m*_ of the wild type by 6.5, 3, and 3°C, respectively, while that in positions 3, 7, and 12 decreased it by −1.5 to −2°C. The slight reduction in *T*_*m*_ probably originated from the stronger lipophilic character of the furyl derivative, relative to T's methyl group [130]. Structures of the intramolecular A(htel-21) and the bimolecular [G_4_T_4_G_4_]_2_ GQs were investigated by using 5-iodouracil (io^5^U) [132].

### 3.2. Derivatives of the *β*-D-Furanose Sugar Moiety

**Figure 9:**
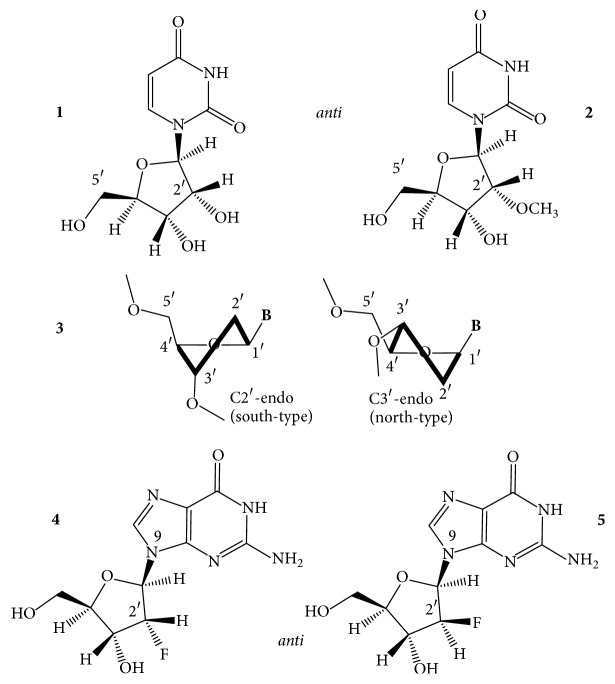
Structures of the* anti* ribo- (**1**) and the 2′-O-methylribouridines (**2**); the C2′-endo (S-type) and C3′-endo (N-type) sugar puckers (**3**); the 2′-fluoro-2′-deoxy*ribo*furanosylguanine (**4**); and 2′-fluoro-2′-deoxy-*arabino*furanosylguanine (**5**).

*Ribonucleosides and *2′*-O-Methyl-ribonucleosides*. Tetramolecular GQs assembling from the ribo hexamer, abbreviated as ug_4_u, were among the first ribo-GQs published. The GQ proved extremely stable in 50 mM KCl [133]. Later, 5-bromouridine-containing tetramolecular structures, [$\underline{br^{5}u}$g_4_u]_4_ [134] and [$\underline{br^{5}u}$gaggu]_4_, were characterized by X-ray diffraction method. The latter GQ contained a g-u octad in addition to guanine and adenine tetrads [135]. The full ribo and the 2′-O-methylribo modifications (Figure 9,** 1**,** 2**) destabilized the monomolecular folds, like the GQs of the TBA, the htel-18 AGG(TTAGG)_3_, and the A(htel-21) GQs, and also bimolecular GQs in Na^+^, such as the [dG_4_T_4_G_4_]_2_, whereas these two modifications stabilized the tetramolecular parallel GQ formed by TG_4_T oligonucleotides in both Na^+^ and K^+^ solutions [1]. Ribonucleosides, due to their thermodynamically preferred* anti* N-glycosidic conformation, originating from the C3′-endo sugar pucker (Figure 9,** 3**), can change the folding topology if incorporated into* syn *dG positions of deoxy-GQs, for instance, as described for the TBA GQ whose antiparallel fold could be changed to parallel and the unimolecular topology to bimolecular [136]. The full ribo-TBA GQ was parallel and was less stable than the deoxy.

Riboguanosines (g) incorporated into* syn* positions of dG1 and dG9 and the* anti* dG4 and dG12 of the two terminal tetrads of the G_4_T_4_G_4_ DNA GQ, which forms a bimolecular basket-type antiparallel GQ with four G-tetrads in both Na^+^ and K^+^ [137], gave rise to N-glycoside conformation-dependent effect on stability in K^+^. Interestingly, substitution of* syn *dGs increased, while that of the* anti* dGs did not change *T*_*m*_ of the wild-type GQ. A single** g** for a G changed the topology. With the exception of GGGGTTTTGGGg, which adopted an antiparallel structure, all hybrids formed parallel, bimolecular GQs [138, 139]. For an NMR study, various GQ-forming sequences like the Myc and htel were systematically modified by single riboguanosine substitutions at* anti *dG positions. Folding topology of most native GQs changed on the formation of DNA-RNA hybrid structures. [140]. Zhou et al. [141] inserted riboguanosine tetrads into the parallel intramolecular TTG_3_TG_3_TTG_3_TG_3_TT, the tetramolecular [TG_3_T]_4_ and [TG_4_T]_4_ GQs. Unexpected destabilizations were observed when the gggg quartets were at the 5′-end of the G stacks in both systems. However, gggg quartets replacing the other dG quartets stabilized the GQ structures.

A single 2′-OMe modification significantly enhanced the thermal stability, by close to 10°C, and also the peroxidase activity of the parallel GQ-forming sequence [B7]-3-0 [142]. Single uridine units were inserted into the three loops of the TBA GQ, separately. The uridine replacing thymidines in the central TGT loop resulted in an increased stability of the antiparallel GQ; however, substitution of thymidines in the TT loops induced destabilization of the TBA GQ [143].

**Figure 10:**
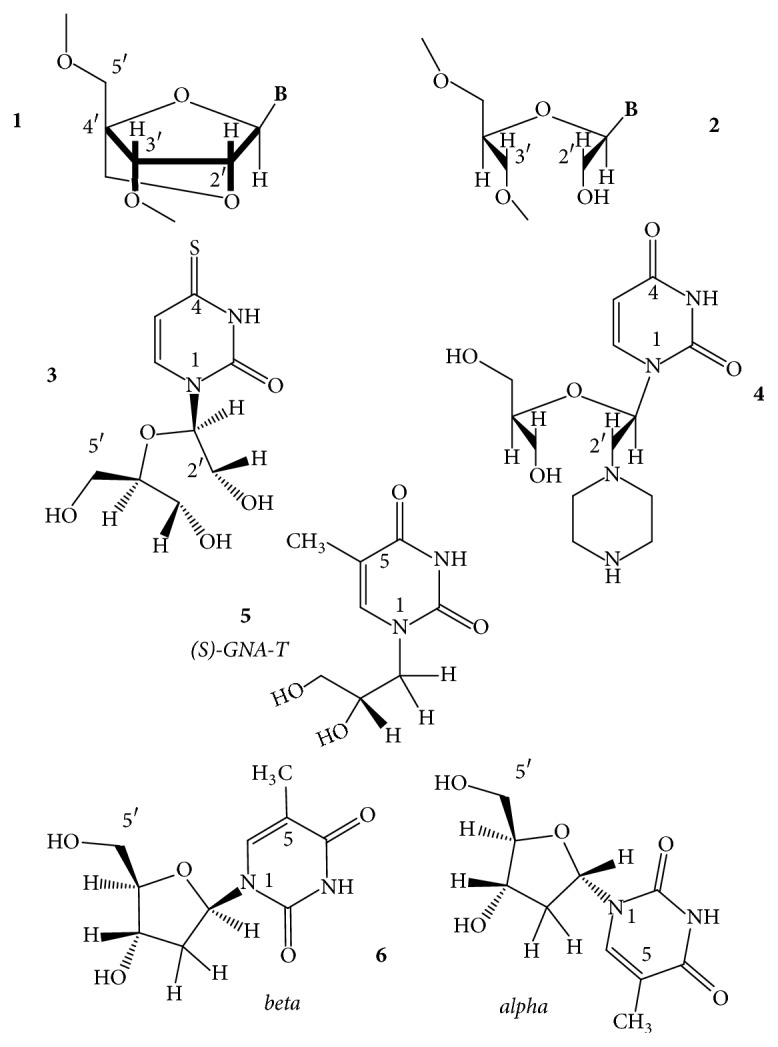
Structures of the locked nucleoside 2′-O-4′-C-methylene-guanosine (LNA), where B stands for a nucleobase (**1**); the unlocked nucleoside 2′-3′-acyclic-ribonucleotide (UNA) (**2**); 4-thio-unlocked-uridine (**3**); 2′-C-piperazino-unlocked-uridine (**4**); an acyclic thymine derivative, (S)-GNA-T (**5**); and the beta- and alpha-thymidines (**6**).

2′*-Fluoro-*2′*-deoxyribofuranosyl- and *2′*-Fluoro-*2′*-deoxyarabinofuranosyl Nucleosides*. Several laboratories have investigated the effect of 2′-deoxy-2′-fluoro-D-arabinonucleotides on GQ structures (fl^2′^araN), where N is any base (Figure 9,** 5**). The antiparallel TBA, the bimolecular [G_4_T_4_G_4_]_2_, and the tetramolecular phosphorothioate backbone-containing anti-HIV [T_2_G_4_T_2_]_4_ were among the first GQ models examined [144]. The energetically preferred N-glycosidic conformation of the fl^2′^araN is* anti*. Incorporation of fl^2′^araG or fl^2′^araT in place of* anti* dG-s of the tetrads or Ts of the loops in the antiparallel or parallel GQs stabilized the structure and maintained the fold of the wild-type GQ [144]. Lech's group [145] also investigated the effect of fl^2′^araG and the 2′-fluoro-2′-deoxyguanosine, fl^2′^dG (Figure 9,** 4**). The deoxy-GQ models were the hybrid-1 TA(htel-21) [101] and the hybrid-2 TA(htel-21)TT in K^+^ [146]. Substitution of the* anti* dG in the middle tetrad by either of the two nucleoside analogs increased the dominance of single folds and also enhanced the thermal stability by about 3°C. However, when all five* syn *dGs were replaced by the analogs, the hybrid forms switched to parallel topology and the thermal stabilities increased by about 10°C, as compared to the stability of the respective wild type. Single riboguanosine (g), arabinofuranosyl-guanine (araG), and 2′-deoxy-2′-fluoro-arabinofuranosylguanine (fl^2′^araG) replaced the dG9 of the 12-mer TAGGGTTAGGGT [147] that builds a mixture of two interconverting folds in K^+^ solution: a dimeric parallel and a dimeric antiparallel GQ [66]. The dG9 was* syn* in the antiparallel form; thus its replacement by the* anti* favoring sugar analogs converted the mixture to the dimeric parallel GQ with the concomitant increase in thermal stability from 41°C to 47–53°C. The fl^2′^araG furnished the greatest stabilization, 12°C [145]. The effect of a single 2′-fluoro-2′-deoxyguanosine (fl^2′^dG), 2′-deoxy-2′-fluoro-arabinofuranosylguanine (fl^2′^araG), and 2′-O-4′-C-methylene-guanosine (LNA) (see next section and Figure 10,** 1**) was analyzed with more than 60 parallel and hybrid GQs [148]. Generally, substitutions of* anti* dGs of the G-tetrads increased the stability of GQs, while substitutions of* syn* positions disrupted the native GQ conformation. The 22-mer**G**GGAT**G**GGACACAGGGGAC**G**GG oligonucleotide folds into a unimolecular hybrid-type GQ in K^+^ solution. 2′-Fluoro-2′-deoxyguanosine (fl^2′^dG), which also favors the* anti* glycosidic conformation, was substituted for the* syn* G1, G6, and G20 (bold, underlined) of the 5′-terminal tetrad. The substitutions changed the polarity of the tetrad. The overall fold, however, did not change and thus created a novel type of GQ in which all four G-columns comprised only* syn* or* anti* dGs: one column with all-*syn* and three G-columns with all* anti* glycosidic linkages [149]. Dickerhoff's group using fl^2′^dG with the GQs of the previously applied 22-mer G_3_ATG_3_ACACAG_3_GACG_3_ and now also with the TT(htel-21)A confirmed the fl^2′^dG-induced conformational perturbations [150]. A single fl^2′^dU was also inserted into the three loops of the TBA GQ. Substitution of thymidines in the TT loops by fl^2′^dU resulted in destabilization of the TBA GQ [143].

Lietard et al. [151] synthesized the first microarrays containing fl^2′^araN and fl^2′^araN/DNA chimeric TBA oligonucleotides to fully map the binding affinity landscape of the TBA GQ. A series of promising fl^2′^ara-modified aptamer candidates were found with *K*_*d*_ values that are significantly lower than that of the unmodified TBA GQ and which adopted highly stable, antiparallel GQ structures. The presence of fl^2′^araT at position T3 not only drastically strengthened the binding but also elevated the thermal stability of the unmodified TBA GQ by 7°C (*T*_*m*_: 47°C). The TBA GQ could accommodate up to ten fluoro-ara nucleotides, in which TBA analogs were also promising aptamer candidates with dissociation constants up to three times lower than that of the wild-type TBA GQ. With these analogs' considerable increase, up to 20°C of GQ stability was observed and stable folding was observed even in the absence of K^+^ ions. The T3 modification apparently preorganized the dinucleotide loop into the proper conformation for interaction with thrombin.

*Locked Ribonucleosides (LNA)*. LNA is the abbreviation for locked nucleic acid. The LNA nucleoside has a modified ribose ring, which is locked in a stable C3′-endo or N-type (RNA-like) sugar pucker by a 2′-O-4′-C-methylene linkage. Thus, the LNA contains a bicyclo-sugar moiety, resulting in a stable* anti* N-glycosidic conformation of the nucleoside (Figure 10,** 1**). The LNA nucleosides increase the stability of duplex and triplex DNAs [152] but the B-type duplex is converted to the RNA-like A-type geometry [153]. LNA oligonucleotides have excellent hybridization properties with DNA and RNA oligomers. The structural impact of LNA in parallel GQs has been characterized by several groups [154, 155]. Nielsen et al. [156] used the telomeric sequence from the* Oxytricha nova*, G_4_T_4_G_4_ for a GQ model. In K^+^ this oligonucleotide forms a dimeric GQ with antiparallel G-columns and diagonal T4 loops [157, 158]. The LNA-substituted sequence G**L**G**L**T_4_G**L**G**L**, where L stands for LNA-G, also formed a dimeric GQ, in which each G-stretch folded back into a V-shaped turn [159] and interacted with each of the three other G-stretches through formation of four G-tetrads. This new GQ folding topology was named the V4 fold. The V4 fold incorporates the features of both parallel and antiparallel GQs in one structure and the remarkable folding topology leads to tetrad steps with both coaligned (between outer and inner G-tetrads) and antialigned (between the two inner G-tetrads) hydrogen bonding, as in the parallel and antiparallel GQs, respectively. CD spectrum of the V4 fold thus displayed signatures of both parallel and antiparallel stacking [156]. In the intramolecular antiparallel, basket-type GQ-forming (G_4_T_4_)_3_G_4_, a single G-LNA or T-LNA reduced the stability of the native GQ in a position-dependent way, most extensively by the G-LNAs [160]. A single G-LNA incorporated at the 3′-terminal G-position of TBA GQ did not change the topology but destabilized the wild-type GQ, by 6°C, from *T*_*m*_ of 52°C to 46°C [161]. The TBA GQ was also the model for G-LNA and T-LNA substitutions at G2 (*anti*), G5 (*syn*, both in the same G-tetrad), and T4 (in a TT loop), T7, and G8 (*anti*, in the TGT loop) positions. G-LNA at position G2 reduced *T*_*m*_ of the unmodified GQ to the largest extent, from 48.1 to 33.5°C in 50 mM KCl, although dG2 is* anti*. Substitution of T7 also reduced *T*_*m*_ by about 5°C, whereas G5-LNA (despite being a* syn* dG position) and G8-LNA (*anti*) increased the thermal stability by 2.6 and 1.9°C, respectively. The T4-LNA GQ was unstable, and hence *T*_*m*_ could not be determined. Thermal stability of the LNA-GQs was more or less inversely related to their antithrombin activity. The substitutions did not change the folding topology of the wild type as Bonifacio et al. reported [162]. The full LNA analog of the TBA DNA did not fold into a GQ, probably due to the rigid nature of the loop position LNA-Ts. The LNA/DNA chimera, in which the tetrad dGs have been substituted by LNA-G nucleotides, did fold into GQ; the CD spectra reflected parallel folding with a peak max. near 260 nm. The hysteresis observed in the melting process referred to a nonunimolecular parallel scaffold. The* anti* LNA-Gs that substituted for* syn* dGs might have induced the formation of the parallel scaffold. The structure was very stable, and the *T*_*m*_ value increased by 20°C relative to the wild type [154].

The htel-12 TAGGGTTAGGGT forms a parallel-antiparallel mixture of dimeric GQs; the latter antiparallel one contains both* anti* and* syn* nucleosides. Substitutions of the natural nucleotides by LNAs, which are restricted to the* anti* form, converted the antiparallel folds into parallel, which contains only* anti* nucleosides. The driving force of the conversion of topology is described as a combination of the C3′-*endo* puckering (Figure 9,** 3**) of the LNA nucleotides and their preference for the* anti* glycosidic conformation. In addition, the LNA-modified parallel GQs are significantly stabilized, by up to 11°C in their *T*_*m*_ value, relative to their DNA counterparts [163]. LNA-G substituting for* anti* dG positions in the parallel-stranded GQ of T_2_G_3_TG_3_TG_3_TG_3_T and the hybrid-1-forming TT(htel-21)A strengthened the GQ, while substituting for* syn* dG positions reduced *T*_*m*_ of the wild type or even disrupted the native GQ structure. Particularly large negative Δ*T*_*m*_ values were described for the parallel-forming GQ, −41 to −45°C, from the 77°C of the native [148].

The tetramolecular parallel GQ [TG_3_T]_4_ was stabilized by LNAs as much as by the ribo substitution [154]. The full LNA GQ showed the smallest hysteresis on reannealing, probably due to the rigid sugar part. The elevated stability was explained by entropy gains. An NMR study of two LNA-modified [TG_4_T]_4_ GQs showed only local structural alterations, which were due to the C3′-endo sugar pucker [155]. The crystal structure of an all-LNA-substituted tetramolecular parallel GQ formed from TG_3_T was determined for the first time by Russo et al., refined at 1.7 Å resolutions. A T-tetrad was formed at the 3′-end [164].

AS1411 is a GQ-forming aptamer capable of selectively entering cancer cells by nucleolin receptor-mediated uptake. Its internalization efficiency highly depends on the chemistry of the oligonucleotides. LNA substitutions were investigated with this GQ [165]. The formation of a GQ from a G-rich 25-mer section of the vascular endothelial growth factor aptamer was highly facilitated by LNA modifications. *T*_*m*_ of ~40°C for the unmodified GQ was raised to ~52°C and ~46°C, respectively, in the two modified structures studied [166]. A series of interesting 3′-end-modified (capped) LNA analogs have been prepared by Kasahara et al. [167], in which the 2′,4′-methylene bridge was replaced by -CH_2_OCH_2_-, -NHCH_2_-, and -CH(Ph)OCH_2_- groups. The bridged nucleosides increased the resistance against nucleases in human serum of the TBA analogs, and the binding abilities were not affected by these modifications. Thermal stabilities were not published. Hotoda's anti-HIV-I oligonucleotide, the tetramolecular parallel GQ-forming TGGGAG's thermal stability, was significantly increased by LNA modifications. The unmodified had a* T*_1/2_ value of 55°C while* T*_1/2_ for TG**GG**A**G** was 75°C, where the bold-underlined Gs were LNA-Gs. The LNA modification highly enhanced the anti-HIV-1 activity of the Hotoda GQ [168]. The U nucleosides of the natural RNA sequence (uuaggg)_n_ forming the TERRA GQ were also replaced by LNA and 2′-O-methyl ribonucleoside analogs to study the protein recognition of the loops ribose moieties. Stability data have not been published [169].

*Unlocked Ribonucleosides (UNA)*. The UNA abbreviation stands for unlocked nucleic acid. In the unlocked nucleosides, the furanose ring's C2′-C3′ bond is missing; it is thus a 2′-3′-acyclic-rN, a ribonucleoside analog (Figure 10,** 2**), which is often abbreviated as, for example, uG or uU. These types of nucleosides are flexible ribo derivatives. The positional effects of single unlocked nucleosides, the 2′-3′-acyclic-rG and –rU, were described with TBA GQs. Replacement of T by uU in loop positions 3, 7, and 12, one in each of the three loops, proved stabilizing for the TBA GQ. uU in the other three T positions destabilized the GQ [170]. Uracil in the loops has been described to stabilize the TBA GQ, contrary to the duplexes, by −0.16 kcal/mol each [1]; thus, with the uU nucleosides, this effect must have contributed to the stabilization. CD spectra of GQs with uU in positions 3, 7, and 12 were the same as that of the unmodified TBA GQ. uU in the other loop positions changed the spectra by significantly lowering the amplitudes and causing band shifts. Incorporation of uG in any tetrad position either destabilized the GQ or hindered the formation of it. The GQ with a uU in position 7 proved to be the only one with more effectiveness in blood clotting than the unmodified GQ. The influence of a single uU and 2′-C-piperazino-uU residues (Figure 10,** 4**) incorporated into several positions of the TBA DNA was studied by Jensen et al. [171], who arrived at very similar conclusions as did Pasternak et al. [170]. The 2′-C-piperazino-uU more efficiently stabilized the GQ structure than the uU and increased the thermal stability of the native TBA GQ by 2-3°C in a position-dependent manner. GQ topology and molecularity were retained. The presence of uU in positions U3, U7, and U12 resulted in the highest stabilization of the GQ. On the contrary, the largest destabilization mounting to −15°C was observed when uU residues were placed in positions U7, G8, and U9. Kinetic studies indicated no strict correlation between thermodynamic stability and the binding affinity to thrombin. Most variants studied bound to thrombin, albeit with decreased affinity related to the wild-type TBA GQ [171]. A double modification study of the TBA with an UNA analog, the unlocked 4-thiouridine, the s^4^uU (Figure 10,** 3**), has also been published. The analog in all possible positions of the three edgewise loops produced negative Gibbs' free energy indicating that at physiological temperature the predominant species were the folded GQ forms. However, most TBA variants were less stable than the unmodified GQ by 0.9–1.0 kcal/mol. s^4^uU at positions 3 and 12 did not influence the thermodynamic stability, whereas, at position 7, it increased it by 0.34 kcal/mol. In contrast, the ribo 4-thiouridine introduced into positions 3, 7, 9, and 12 stabilized TBA GQ by 0.31–0.53 kcal/mol and was usually more stabilizing/less destabilizing than the s^4^uU. The modified TBAs retained the antiparallel GQ topology of the native GQ. Thrombin clotting time studies revealed that TBA modified with s^4^uU at position 7 possessed high anticoagulant activities and the modified aptamer was a potent inhibitor of fibrin-clot formation [172]. Unlocked uA, uT, or uC was substituted for the single-base diagonal loop and uG for tetrad Gs in the GQ of G_3_T_5_G_3_A(or T,C)G_3_T_5_G_3_. The loop (A, T, or C) modifications stabilized the GQ by 3–7°C in *T*_*m*_, and the uA had the greatest effect. Stabilization was explained by the flexibility of the unlocked nucleoside, which could ease the tension that might exist in a single-base diagonal loop. Contrary to the loop substitutions, the uGs led to significant destabilization of the GQ. The uG in the middle tetrad caused the largest effect, and Δ*T*_*m*_ was −17.6°C. Flexibility of the sugar moiety did not prove to be a beneficial structural motif in the G-tetrads. The unlocked nucleosides caused transition from the antiparallel to hybrid-type fold in some of these GQ analogs [173]. Thymine glycol, also an acyclic analog of dT, (S)-GNA-T (Figure 10,** 5**), was substituted for loop T position of the TBA GQ. The analog destabilized the TBA in positions in T4,T9 by 9–13°C, and stabilized it in T7 by 5.2°C. With double substitutions in T7,9, T3,12, and T4,T13, the TBA GQ was destabilized by 2–12°C [94]. Aaldering et al. [57] reported on the impact of uU and the 3′-amino-uU on the structural dynamics and stability of TBA GQ by substituting them for the (nonstacking) loops T3, T7, and T12. While the uU enhanced the thermal stability of the unmodified GQ (*T*_*m*_: 49°C) at all three T positions, by 2–5°C, the 3′-amino derivative decreased it by 2–7°C. As the amino group replaced the 3′-OH, the internucleotide linkage at this site was 2′-5′ instead of the natural 3′-5′ (this modification of the backbone is named* iso*DNA; see also later). The authors suggested that the altered backbone caused the destabilization effect with the 3′-amino modifications. On the other hand, none of the loop substitutions changed the topology of the native TBA GQ (CD peak max. at 295 nm and negative peak at 265 nm), and both the unlocked uridine analogs improved the thrombin clotting time [57].

*α-Anomeric Sugars*. *α*-Nucleosides differ from the natural *β*-ones by the inversion of the configuration at the C(1′) anomeric position of the furanose ring (Figure 10,** 6**) (for a review, see [174]). The T_2_G_4_T_2_ that assembles into a parallel-stranded tetramolecular GQ was found to be an inhibitor of HIV infection, especially if the phosphodiesters internucleotide linkages were replaced with P-S linkages (see Section 3.3), and its IC_50_ value was 0.3 *μ*M [175]. When the same oligodeoxynucleotide was built from *α*-nucleosides, the *α*(T_2_G_4_T_2_), also with P-S linkages, a similar anti-HIV activity was observed, and the IC_50_ value was 0.5 *μ*M [176]. Since *α*-oligonucleotides are nuclease-resistant [174], it was claimed that the P-S backbone is mechanistically required for antiviral activity of this oligonucleotide. It was, therefore, suggested that PS-ODN interacts with the highly cationic V3 loop. *α*-Nucleosides, which cannot adopt* syn* glycosidic conformation [177], were substituted for Ts of the loops and dGs of tetrads of the native TBA GQ. Depending on the location of substitution, the GQ was either stabilized or destabilized by the *α*-Ts in the loops. Significant stabilization was observed for the anomeric modification of TT loops at T4 and T13 (Figure 2). The T-substitutions did not change the antiparallel topology. The *α*-dGs, however, either prevented the GQ assembly or induced rearrangement of the topology. Replacement of all natural nucleotides with the anomeric ones also resulted in random coil. The anticoagulant properties of the chimeric aptamers were retained only with intact TT loops. On the contrary, modification of the TGT loop only substantially increased the nuclease resistance of the chimeric aptamer without notable disturbance of its anticoagulant activity [178].

**Figure 11:**
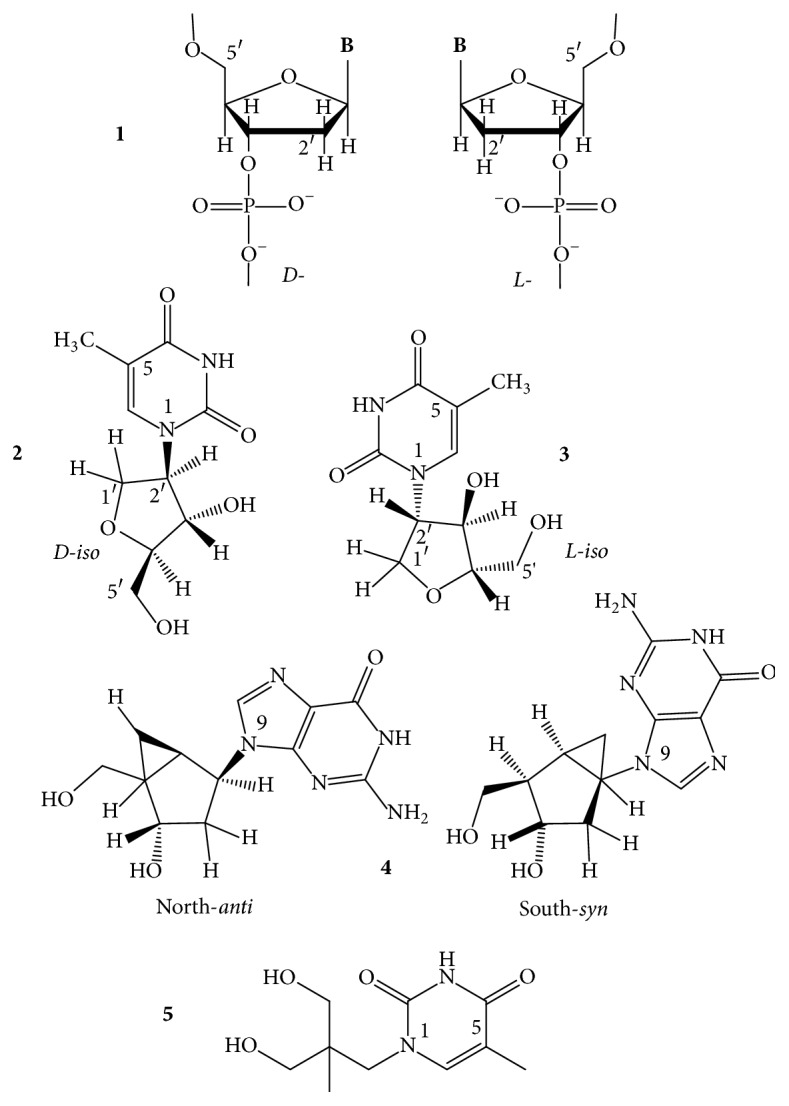
The mirror image D- and L-2′-deoxynucleosides (**1**); the D- (**2**) and L-*iso*-thymidines (**3**); the* anti* north- and the* syn* south-bicyclo[3.1.0]hexane-dGs (**4**); and an acyclic thymine analog, the N^1^-(3-hydroxy-2-hydroxymethyl-2-methylpropyl)-thymine (**5**).

*L-Nucleosides*. L-Nucleosides (Figure 11,** 1**) are mirror images of the D-counterparts. Zintevir is a 17-mer DNA that assembles into a GQ of intramolecular parallel topology due to the single-base loop(s). This GQ shows strong anti-HIV-1 activity. The GQ from its L-sugar enantiomer building blocks, the L-GTGGTGGGTGGGTGGGT, showed comparable antiviral activity to Zintevir. Its CD spectrum was a mirror image and completely symmetrical to that of the D-GQ and thermal stability of the L-analog was very similar to that of the D [179]. The L-analog of the tetramolecular parallel GQ-forming TG_4_T was also synthesized later [180]. CD spectrum of L-[TG_4_T]_4_ was again the inversion of the D-scaffold and thermal stabilities (*T*_1/2_) were identical within the experimental error, 57°C for the D- and 58°C for the L-structure. Thermal difference UV spectra, diagnostic of GQ formation, were nearly superimposable. Heterochiral or D/L-oligonucleotide chimeras based on TG_4_T have been prepared by Antonella Virgilio et al. [181]. These could form either right-handed D- or left-handed L-GQs, depending on the composition and sequence. The T_D_G_D_G_L_G_L_G_D_T_D_ formed left-handed topology even if it was mainly composed of D-nucleotides. L-Nucleotides were also incorporated into the 15-mer TBA DNA. All TBA sequences that contained various numbers of L-nucleotides were able to fold into the chair-type antiparallel GQs, similar to the wild type. Thermal stability of the full-L GQ was the same as that of the D; only the heterochiral structures were generally less stable than full chirals [182]. L-Nucleotides were also combined with polarity inversion sites. Virgilio et al. [183] prepared the heterochiral oligodeoxynucleotides 5′-T_D_G_D_G_D_-3′-3′-G_L_G_L_T_L_-5′ (L33) and the 3′-T_D_G_D_G_D_-5′-5′-G_L_G_L_T_L_-3′ (L55). Both formed GQ structures and data indicated the presence of enantiomeric left- and right-handed GQ helices. In the case of L55, NMR experiments pointed to an unusual equilibrium between them. TBA sequence was also derivatized, like the 5′-G_L_G_L_-3′-3′-TT-5′-5′-G_L_G_L_T_L_G_L_T_L_G_L_G_L_-3′-3′-TT-5′-5′-G_L_G_L_-3′. Interestingly, all modified oligodeoxynucleotides folded into the two-tetrad, chair-like antiparallel topology. Thermal stabilities depended on the position of the L-nucleotides and the inversion sites [184]. Aviñó et al. substituted the TBA thymines with L-Ts in loop positions 3, 4, and 7, and all destabilized the GQ by 0.7 to 4°C [94].

GQs and i-motif (C-)GQs have also been built from *β*-L-*ribo*nucleotides. Thermal stability of the *β*-L-r(AGGAGGAGGAGGA) GQ and the *β*-L-r(C_3_UC_3_U_4_C_3_UC_3_) i-motif in 100 mM KCl and pH 7 and pH 3.4, respectively, was the same or very similar to those of the *β*-D-ribo, that is, to the natural GQs, near 73°C and 33°C, respectively [185]. A recent paper also dealt with anomeric L-nucleic acids by examining the structural features of a mixed L-RNA/L-DNA GQ aptamer [186]. When L-nucleotides were incorporated into a DNAzyme sequence, which could fold into an intramolecular L-GQ, the resulting L-DNAzyme exhibited peroxidase catalytic activity [187].

*Isonucleosides*. Instead of the natural C1′-N1 N-glycosidic linkage, the C2′-N1 linkage-containing* iso*thymidines (Figure 11,** 2**,** 3**) were introduced into the loop section of the TBA DNA, in which the* iso*nucleosides were also in either the natural D or the mirror image L stereo configuration, the D- and L-isothymidines [188]. Incorporation of D-*iso*T into positions T3 and T12 of the two TT loops and T7 of the TGT loop (Figure 2) increased the thermal stability of native TBA GQ by 0.9 to 5.4°C. The mirror image L-*iso*T in positions of T3, T12, and T9 (TGT loop) also increased the stability by 2.5 to 5.1°C. Substitution of all other T positions by either D- or L-*iso*T resulted in significant destabilization of the scaffold, by −3.3 to −17.5°C. Mixed double modifications were also tested in this study from which a few also stabilized, while others destabilized the native TBA GQ [188].

*Other Modifications of the Sugar Moiety*. Besides LNA, another conformationally rigid sugar analog of dG, the* syn* and* anti*, north- and south-bicyclo[3.1.0]hexane-dGs (Figure 11,** 4**) were substituted for G14 and G15 of the TBA GQ [189, 190]. Substitution of position 14 did not change the stability and topology of the wild-type TBA GQ; substitution of position 15, however, caused a strong destabilization. Coppola et al. [191] substituted TBA's thymidines, one at a time, with acyclic thymine analogs, namely, N^1^-(3-hydroxy-2-hydroxymethyl-2-methylpropyl)-thymine (Figure 11,** 5**), a sort of unlocked nucleotide. Nearly all TBA analogs were able to fold into similar GQ structure as the native DNA did. Substitution of loops T3, T7, and T12 by the acyclic-Ts slightly stabilized, by 1–4°C, the GQ, relative to the unmodified TBA, whereas that of T4 and T13 slightly destabilized, by 3°C, and that of the T9 significantly reduced the thermal stability of the unmodified TBA, from 50°C to 39°C [191].

*Multiple Modifications of the Sugar Part*. When hemin interacts with certain GQs, the complex will exhibit peroxidase activity. These hemin-binding GQs have recently emerged as important peroxidase mimicking DNAzymes and have been utilized in bioanalytical applications. Li et al. [192] studied the effect of multiple chemical modifications of the sugar part: 2′-O-methyl-, L-nucleotides and LNAs; furthermore, the backbone was also modified with phosphorothioates (P-S). Three GQs with known high peroxidase activity were selected for the experiments, the PS2.M, GTG_3_TAG_3_CG_3_T_2_G_2_, the c-Myc, TGAG_3_TG_4_AG_3_TG_4_A_2_, and the EAD2, CTG_3_(AG_3_)_3_A. Results indicated that the 2′-O-methyl modification facilitated the formation of perfectly compacted parallel structures with the highest thermal stabilities among the unmodified and modified GQs and with significantly promoted peroxidase activity of GQ based DNAzymes. The LNA, L-, and the P-S backbone modifications reduced the stability of wild types [192].

GRO29A is a GQ-forming, growth-inhibitory oligonucleotide, whose sequence is T_3_G_2_TG_2_TG_2_TG_2_T_2_GTG_2_TG_2_TG_2_TG_2_ [193]. Several sugar and backbone-modified analogs of GRO29A have been prepared by incorporating 2′-O-methylribo-, mixed 2′-deoxy, and 2′-O-methylribo nucleosides and backbone-modified P-S analogs. The full P-S, the 2′-O-methyluracil (RNA) 2′-deoxyguanosine-containing chimeric and the full 2′-O-methylribo analogs increased the thermal stability of the GQ of the 3′-aminoalkyl-modified GRO29A, while the other analogs decreased it or no thermal profiles were detected. The P-S and chimeric analogs were found to significantly inhibit proliferation of a number of tumor cell lines, but the 2′-O-methyl RNA analog had no significant effect. Based on molecular modeling, it was concluded that the inactivity of the latter was due to the differences in the groove structure of the GQ, compared to the other's [194].

The effects of multiple substitutions of the TBA DNA by LNA and 2′-O-methyl analogs of G (LNA-G and om^2′^G) on the formation and topology of the TBA GQs have been studied. Results showed that when two or more Gs were substituted with LNA-G or om^2′^G, the 15-mer TBA oligonucleotide remained unstructured in 50 mM K^+^. The native TBA GQ was unstructured in 50 mM Ca^2+^, and interestingly, four or more LNA-G or om^2′^G substitutions induced the formation of parallel GQs, which were dimeric forms [195].

*Chain Terminal Modifications*. “Sweetening” the TBA GQ, that is, attaching various 5′-terminal sugar derivatives to the 15-mer TBA DNA, led to the reduction of the thermal stability of the native GQ by 4.5 to 10.1°C. The sugar moieties were connected to the 5′-dG through a phosphodiester group, for example, the *β*-D-glucose-C_2_-OPO_2_-5′-TBA. *β*-D-Galactose, -maltose, -lactose, -cellobiose, and *β*-L-fucose were also appended. Compared to the HO-C_2_-OPO_2_-derivatized TBA GQ, not the native TBA GQ, there was a slight increase in the *T*_*m*_ values, 0.7 to 2.8°C, with the added sugar moieties. Attaching carbohydrates to the 5′-end of the telomeric oligonucleotide of TAGGGTTAGGGT could alter the folding topology of the parent GQ. The sugar moieties can well stack with the 5′-G-tetrad [196]. Four cationic amino acids, lysine, ornithine, homo-arginine, and arginine, were covalently attached to the 3′-end of the tetrahymena telomeric repeat DNA sequence TG_4_T [197]. The cationic residues did not affect the formation of parallel tetramolecular GQ structures, and the 3′-conjugations increased the thermal stability of [TG_4_T]_4_ by 3 to 6.4°C in *T*_*m*_ in 0.11 M Na^+^ [197]. Thermal stabilization by several other terminal modifications of tetramolecular GQs has been reviewed by Doluca and coworkers [4] and more recently by Prokofjeva et al. [15] who analyzed the GQ-forming chain end-modified oligonucleotide aptamers, primarily the Hotoda TGGGAG analogs, in connection with their anti-HIV activity. The anti-HIV activity of GQs is exerted via preventing the viral entry into the target cells by binding to the gp120 surface glycoprotein of the virus and by targeting the viral integrase or the viral reverse transcriptase within the HIV, as it was reviewed by Musumeci et al. [16]. The 5′-terminal modifications were the dimethoxytrityl, 3,4-dibenzyloxybenzyl, tert-butyldiphenylsilyl, and other aromatic groups. 3′-End modifications also enhanced the aptamer activity, such as the 2-hydroxyethylphosphate, glucose, or the mannose moieties. LNA-G at certain tetrad positions also resulted in potent activity with increased thermal stability of the GQ. No strict correlation was found between the stability and the aptamer activity of the GQs. Terminal modifications, especially the hydrophobic groups, can increase the thermal stability of GQs. The backbone modifications, such as the thiophosphoryl (P-S) internucleotide linkages, increased the resistance to cellular nucleases, which is important for the aptamer activity [15].

### 3.3. Phosphodiester and Sugar-Phosphate Backbone Analogs

**Figure 12:**
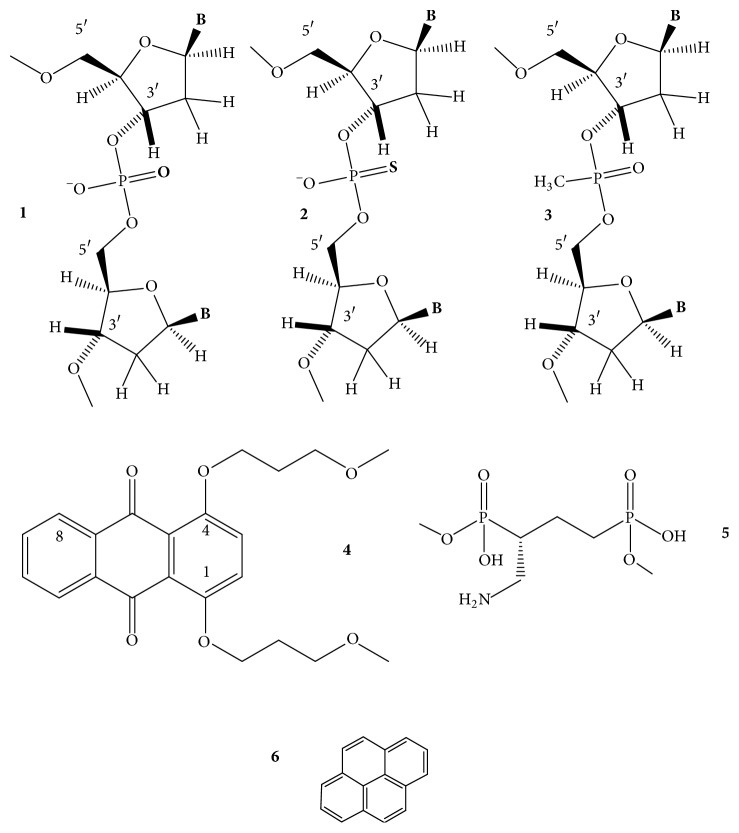
2′-Deoxydinucleotides connected by the natural phosphodiester (P-O) (**1**); the thiophosphoryl or phosphorothioate (P-S) (**2**) and the nonionic methylphosphonate (P-Me) linkages (**3**); the 1,4-disubstituted dihydroxyanthraquinone linker (**4**); the acyclic (R)-4-aminobutane-1,3-diol phosphodiester backbone (**5**); and the pyrene molecule used for loop (**6**).

*Phosphorothioates (P-S)*. A sulfur atom replaces one of the nonbonded oxygen atoms of the phosphodiester (P-O) internucleotide linkage (Figure 12,** 1**) in the phosphorothioate (P-S) linkage (Figure 12,** 2**). The sulfur can be in R_P_ or S_P_ stereo configuration, bringing in the chirality problem. In most cases, however, the isomers are not separated after the synthesis. Sulfur has a larger size than the oxygen and is less hydrophilic. If the P-O linkages are replaced with the random stereo mixture P-S linkages in a DNA or RNA strand, its duplex with an unmodified complementary strand is thermodynamically destabilized relative to the full P-O duplex. If the pure stereo full-R_P_ or full-S_P_ isomeric strands are compared, the negative effect of S_P_ isomers is larger than the full-R_P_ strands in their duplexes [198]. However, the P-S strands become highly resistant against cellular nucleases, and this property made the P-S oligonucleotides the first generation of therapeutically applicable antisense oligonucleotides, as reviewed by Eckstein [199]. As for the GQs, when all P-O linkages were replaced by P-S in the tetramolecular parallel [TG_4_T]_4_, in the 12-mer bimolecular [G_4_T_4_G_4_]_2_ GQs in Na^+^ and in the intramolecular antiparallel TBA GQ in K^+^, the GQs were all destabilized. The P-S substitution of the TBA was studied in more detail by Zaitseva et al. [200] and Prokofjeva et al. [15]. The CD spectrum of the unmodified TBA GQ was not changed by P-S modifications. Presence of a single P-S bond between two G-quartet planes (the G-G bond) led to a significant decrease in GQ thermostability, from 52°C to 45°C in 100 mM K^+^. On the contrary, modifications in the loop sequences (T-T bonds) either did not alter or stabilized the structure [15].

Interestingly, the P-S substitution had no effect in Na^+^ and increased the stability in K^+^ of the intramolecular GQs built from the 18-mer AGG(TTAGG)_3_ and the AG_3_(TTAG_3_)_3_ [1]. Another sequence, the T_2_G_4_T_2_, forms a parallel-stranded GQ, which has antiviral activity. The tetramolecular structure binds to the human immunodeficiency virus envelope protein gpl20 at the V3 loop and inhibits both the cell-to-cell and virus-to-cell infection. The P-S analog of T_2_G_4_T_2_ was found to be a more potential inhibitor of HIV infection,* in vitro* [175, 176]. Site-directed spin labeling technique was used for investigating GQ conformations by Zhang et al. [201]. Double nitroxide labels were attached to the sulfur atom of a P-S linkage at G3 and G15, the G4 and G15, and the G9 and G15 of the A(htel-21) oligonucleotide. The labels only marginally impacted the folding feature of the GQs and could adequately distinguish between different topological conformations of the A(htel-21) GQ.

*Inversion of Polarity Sites*. In natural nucleic acids, the nucleotides are connected via 3′-5′ phosphodiester linkages. When this is changed to 3′-3′ or 5′-5′ linkages, it is called inversion of polarity. In GQ-forming oligonucleotides, such sites can be introduced at three different locations: in non-G-tracts, in G-tracts, and between a non-G-tract and a G-tract. The polarity inversion modifications in GQs have been reviewed by Virgilio et al. [9]. The inversion polarity site-containing GQs, a new class of GQs, have been introduced in 2005, and the first such structures contained 3′-3′ or 5′-5′ inversion sites in the G-tract [202]. Later, a library of 14 inversed-polarity site-containing TBA GQs have been prepared: seven with 5′-5′ and seven with 3′-3′ inversions. The former started with the 3′-G5′-5′GTTGGTGTGGTTGG-3′ and continued up in the sequence up to 3′-GGTTGGT5′-5′GTGGTTGG-3′. The latter group started in 5′-G3′-3′GTTGGTGTGGTTGG-5′ and continued up to 5′-GGTTGGT3′-3′GTGGTTGG-5′. The structures were characterized by NMR spectroscopy. The 5′-5′ inversion site in the G-tract prevented the formation of stable GQ structures [203]. Another study showed that, among the T5′-5′T series, the 3′-GGT5′-5′TGGTGTGGTTGG-3′ sequence folded into an unusual structure adopting three strands parallel to each other and only one strand oriented in the opposite manner. This modified TBA was more stable than its unmodified counterpart and showed a high thrombin affinity. *T*_*m*_ of the wild-type GQ was 53°C and that of the inversed-polarity analog was 57.9°C [204, 205]. GQ of the 3′-TG5′-5′GGGT3′ (QS55) and the 5′TG3′-3′GGGT5′ (QS33) formed different topologies. NMR analysis revealed that the latter formed a parallel-like fourfold symmetric GQ, while the former possessed twofold symmetry and was characterized as a tetramer antiparallel GQ embedded between two parallel tracts. The inversed-polarity-containing GQs had reduced thermal stability relative to the native fourfold GQ. *T*_*m*_ values of 65°C, 47°C, and 53°C were measured for the [TGGGGT]_4_, the [5′TG3′-3′GGGT5′]_4_, and the [3′TG5′-5′GGGT3′]_4_, respectively [206]. NMR and CD studies of two polarity-inversed GQs, named Q33 and Q55, formed by the oligodeoxynucleotides 5′-TGG3′-3′GGT-5′ and 3′-TGG5′-5′GGT-3′, respectively, had different arrangement of the glycosidic angles of the residues, which led to a different symmetry and different physical properties of the two GQs. Both GQs assumed right-handed conformation. The Q33 had all G residues in* anti* glycosidic conformation, while the Q55 had one G-quartet with all-*syn* residues. Interestingly, the Q55 was more stable by 25°C than the natural counterpart, whereas Q33 was a less stable structure (*T*_*m*_ = 52°C) [207]. Inversed-polarity sites were also introduced into the TG_3_T oligonucleotide, which folds into [TG_3_T]_4_ in K^+^ solution [208]. The presence of these unnatural sites did not hinder the formation of tetramolecular GQs and in some cases increased the thermal stability of the unmodified one. *T*_*m*_ of the GQ formed from the native sequence TGGGT was 45°C, that of 5′T3′-3′GGGT5′ was 44°C; that of 5′TG3′-3′GGT5′ was 49°C; that of 3′T5′-5′GGGT3′ was 45°C; and that of 3′TG5′-5′GGT3′ was 72°C [205]. Virgilio's group has also studied 5′TGGGGT3′ analogs that contained two or three 3′-3′ or 5′-5′ inversion sites in the G-run, namely, 5′TG3′-3′G5′-5′GGT3′, 5′TG3′-3′GG5′-5′GT3′, 5′TGG3′-3′G5′-5′GT3′, 5′TG3′-3′G5′-5′G3′-3′GT5′, and 3′TG5′-5′G3′-3′G5′-5′GT3′ [209]. The modified sequences contained either no or only one natural 3′-5′ linkage in the G-tract; notwithstanding, all analogs formed stable tetramolecular GQs. Results showed that the 3′-3′ or 5′-5′ inversion sites affected the glycosidic conformation of dGs and, consequently, also base stacking. This study allowed the authors to depict some generalizations concerning strand arrangements and the glycosidic conformational preference of residues adjacent to inverted polarity sites [209]. The inversion sites could increase the resistance of the TBA GQs against degradation by cellular 3′-exonucleases. Eight TBA analogs have been examined, which contained the inversion site at the 3′-end of the oligonucleotides that also contained an added natural base at the 3′. Some of them contained also a 5′-inversion site also with an added nucleotide. A few TBA analogs not only were much less sensitive towards various exonucleases but also were active aptamers and had elevated thermostability. The 5′-G_2_T_2_G_2_TGTG_2_T_2_G_2_-3′-3′-A was the most stable with a Δ*T*_*m*_ of 12°C, and the A-5′-5′-G_2_T_2_G_2_TGTG_2_T_2_G_2_-3′-3′-T was the least stable with a Δ*T*_*m*_ of −1°C, relative to the stability of the unmodified GQ. The modifications did not change the antiparallel, chair-type conformation characterizing the wild type [210].

Polarity inversion-site containing GQs proved more resistant to exonucleases than the wild types [210, 211], and some of them also showed antiproliferative activity [211]. GQ analogs have also been built from (TG_2_T)_4_, (TG_3_T)_4_, and (TG_4_T)_4_ that contained one 3′-3′ and two 5′-5′ inversion sites. The modified (TG_3_T)_4_ and (TG_4_T)_4_ formed very stable monomolecular parallel GQ structures characterized by three side loops containing the inversion of polarity sites. Both contained an all-*syn* G-tetrad, while the other dGs adopted* anti* glycosidic conformations. Both GQs showed remarkable antiproliferative activity against cancer cell lines [211]. Oliviero et al. [212] published on polarity site inversion GQs with the aim of obtaining structurally homogenous DNA G-wire nanostructures, using 5′-CGGT-3′-3′-GGC-5′ sequences. An NMR study performed by Šket et al. on the tetramolecular GQs formed by TG_3_T and its analogs containing a 5′-5′ or 3′-3′ inversion of polarity site, namely, the 3′TG5′-5′G_2_T3′, 3′T5′-5′G_3_T3′, and the 5′TG3′-3′G_2_T5′, revealed that the parallel GQs had distinct cation-binding preferences [213]. When not only one or more inversion sites are introduced into an oligonucleotide but also the whole sequence is reversed from the 5′-3′ direction to 3′-5′, Marušič and Plavec [214] introduced the term sequence inversion or sequence. G-rich oligonucleotides of this full sequence inversion could assemble into GQs; however, both the thermal stability and the number of structures formed changed, as it was described for the natural 5′-3′ 27-mer G_3_TAG_3_CAG_4_ACACAG_3_TAG_3_, a noncoding segment of the human papilloma virus (HPV) type 52, with its two truncated sequences. The reversed sequence of the 27-mer, illustrated as 5′-3′, was the G_3_ATG_3_ACACAG_4_ACG_3_ATG_3_. For the three reversed sequence-GQs, the *T*_*m*_ values were lower by 1 to 7°C in K^+^ and 3 to 7°C in Na^+^. The inverted sequences showed completely different folding preferences from what the natural sequences did [214].

Another example for the inversed polarities is with hexaethylene-glycol (HEG). A nonnucleotide bridge connects two TG_3_AG oligonucleotides, whose connection was built with inversed polarities: 5′-TG_3_AG3′-p-HEG-p-3′GAG_3_T-5′ and the 3′-GAG_3_T5′-p-HEG-p-5′TG_3_AG-3′. In K^+^ solution, the molecules formed tetramolecular parallel GQs where the two HEG bridges formed two loops. The wild-type tetramolecular parallel GQ of TG_3_AG had a *T*_*m*_ of 41.5°C. The HEG GQs had much higher thermal stability: the first analog 76.0°C and the second 75.5°C. The conjugated GQs exhibited elevated resistance in human serum and high or moderate anti-HIV-1 activity with low cytotoxicity. As a result, these conjugated hairpins represent the first active anti-HIV-1 bimolecular GQs based on the TG_3_AG sequence [215]. A doubly modified GQ containing polarity inversion has also been described: L-residues and inversion of polarity sites have been introduced into five TBA DNAs by Esposito et al. GQs of the all-L-TBA oligonucleotides as well as the all-L minus the first and third TT loops, which remained D-thymidines, folded into left-handed GQs, and, interestingly, thermal stability of the wild type was retained in both modified structures. Two mixed L- and inversed-polarity site sequences also formed left-handed GQs and their stabilities changed depending on the site of modification. The TBA analogs containing L-residues and inversion of polarity sites lost the anticoagulant activity but gained antiproliferative properties against two cancer cell lines [216]. In an NMR study, Esposito et al. used inversed-polarity-d(TGGGT)s, which also contained an AP (abasic) site, and revealed interesting changes of the* anti* and* syn* glycosidic conformation in the G-tetrads of the tetramolecular parallel GQs [217].

*Nonnucleotide Backbone*. 1,4-Dihydroxyanthraquinone and 1,8-dihydroxyanthraquinone linkers (Figure 12,** 4**) were substituted by Gouda et al. [218] for the loop T nucleotides of the TBA GQ. Single substitutions generally reduced the stability of native TBA (*T*_*m*_ 50°C in 100 mM K^+^). On the other hand, double substitutions by either linker led to stabilizations by 4 to 26°C. The latter was achieved with the 1,4-disubstituted anthraquinone linker in positions T4 and T13 in the two lateral TT loops. The modifications retained the antiparallel conformations of the native TBA GQ. Majority of the anthraquinone-modified TBA GQs showed decreases in the clotting times, compared to TBA. The aptamers containing the 1,8-linker at G8 or T9 in the TGT loop had improved anticoagulant activity [218]. Gouda et al. [219] also replaced the TT and TGT loops of the TBA GQ with novel 1,5- and 2,6-disubstituted anthraquinones. Single substitutions destabilized, while anthraquinones in two TT loops led to 1–18°C increase in *T*_*m*_ of the parent GQ without changing the original topology. TBA nucleotides have also been substituted with acyclic (R)-4-aminobutane-1,3-diol phosphodiester backbone (Figure 12,** 5**). Substitution of T7 resulted in a slight increase in *T*_*m*_ with no effect on conformation. Double substitutions of G5G6, or multiple T positions, resulted in significant decreases in the *T*_*m*_ and the Δ*G*_310_ values [220]. One, two, and all three TTT loops were replaced by pyrene molecules (Figure 12,** 6**) of the 21-mer G_3_(T_3_G_3_)_3_ oligodeoxynucleotide by Rajagopal and Hariharan [221]. In 100 mM KCl, the thermal stability of parent antiparallel/hybrid GQ (positive max. around 290 nm with a strong shoulder/peak near 260 nm, negative max. close to 240 nm in its CD spectrum) of 70.2°C was not changed by a single pyrene backbone, *T*_*m*_ 69.9°C; however, the stability was increased to 74.5°C and 87.2°C for the two and three pyrene backbone-containing GQs, respectively. Concurrently, CD spectra showed the formation of scaffolds with elevated content of parallel strands by the increase of the 260 nm peak. In the deep eutectic solvent of 1 : 2 choline chloride-urea containing 100 mM KCl, mimicking viscous biological conditions, the *T*_*m*_ values decreased from 68.8°C to 60.9, 40.8, and 36.6°C for the one, two, and three pyrene backbone-containing GQs, respectively. CD spectra of the unmodified GQ showed parallel folding, to which the pyrene-modified GQs converted from hybrid form with increasing pyrene content [221].

## 4. Synthetic Nucleotides That Only Destabilized a GQ Structure

### 4.1. Destabilizing Base Derivatives

**Figure 13:**
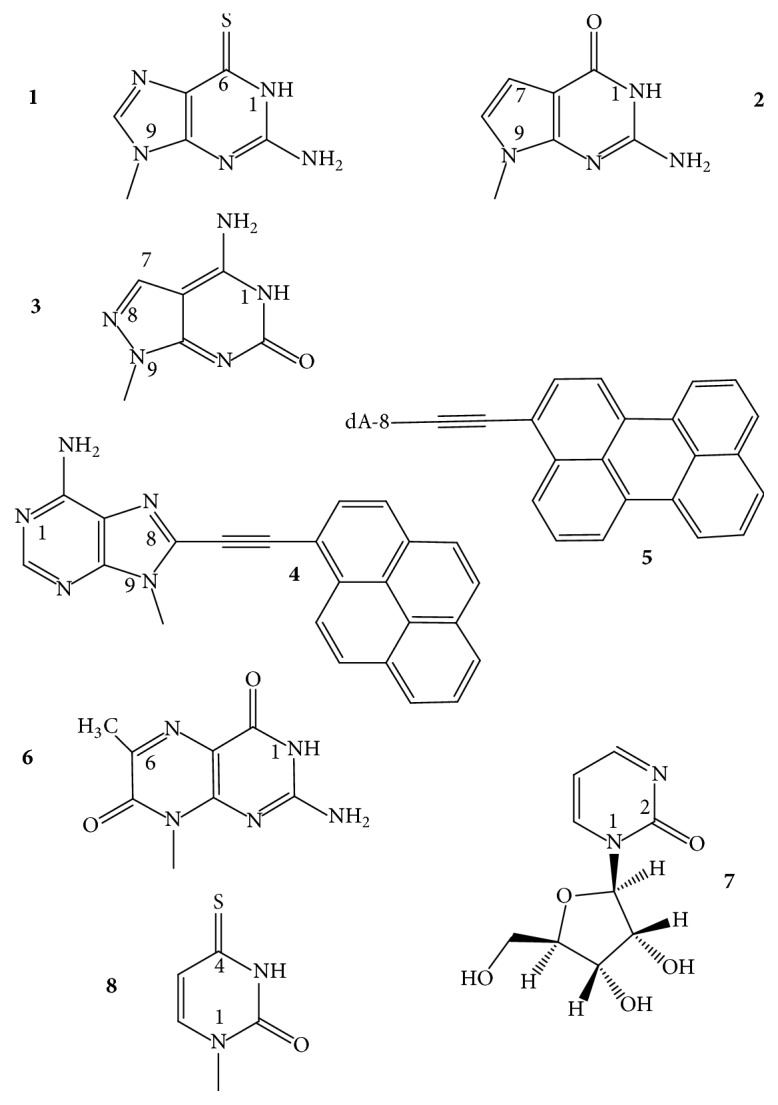
Structures of 6-thioguanine (**1**); 7-deazaguanine (**2**); 8-aza-7-deaza-*iso*guanine (**3**); pyrene (**4**) and perylene (**5**) tethered to C8 of guanine; 6-methyl-isoxanthopterin (**6**); zebularine or 2-pyrimidinone-riboside (**7**); and 4-thiouracil (**8**).

*6-Thioguanine (s* ^6^ *G) and 6-Mercaptopurine (s* ^6^ *Pu)*. The medication 6-thioguanine (Figure 13,** 1**), used against leukemia and ulcerative colitis, has also been introduced into various GQ-forming sequences, such as the TG_4_T, TG_5_T, TBA, and the htel repeats. In K^+^ the s^6^G in none of the G positions altered the characteristic CD spectra of [TG_4_T]_4_ and [TG_5_T]_4_ but sharply destabilized the structures, as Gros et al. reported [222]. s^6^G was also incorporated into position 13 of the two-tetrad-forming (htel-21)T. The GQ contained a br^8^G at position 7, as well, and the effect of s^6^G on stability was not separately discussed. The overall topology was not changed by s^6^G [77]. However, s^6^G inhibited the formation of TBA's antiparallel, two-tetrad, chair-type GQ when it substituted for G2 in G$\underline{s^{6}G}$T_2_G_2_TGTG_2_T_2_G_2_ or in G_2_(TU)_2_G_2_(UT)_2_G_2_U_2_T_2_G_2_ [223] although the U base alone is not an inhibitor of GQ formation, for example, in G_4_(T_4_G_4_)_3_ [224], which is only one of the many cases known [5, 8]. The destabilizing effect of s^6^G was also studied by molecular dynamics calculations [225]. Destabilizing effect of a new purine analog, 6-mercaptopurine, has been described by Radhika et al. [226] based on MD simulations of the GQ structure of (TGGGGT)_4_ containing a single analog that also caused local distortion of the fold.

*6-Selenoguanine (se* ^6^ *G)*. Molecular dynamics simulations of the TBA GQ showed that although one nucleotide of se^6^G was tolerated in the scaffold, the thermodynamic stability was reduced accompanied by conformational alterations. Two or more such mutations prompted unfolding of the scaffold due to steric clashes in the interior channel of the GQ, which led to the release of the central K^+^ ion and to the disruption of the structure [227].

*7-Deazaguanine (c* ^7^ *G)*. In GQ models, the 7-deazaguanine (Figure 13,** 2**) was first mentioned in 1992 demonstrating that “regular” GQ structures cannot be formed if c^7^G replaced a G in the core [228]. Biological functions were also impaired by this analog, as it was shown when it substituted individually for each G-position of the 15-mer TBA DNA [19, 20]. The TBA aptamer binds to and inhibits thrombin. 7-Deazaguanosine in each of the eight tetrad positions significantly reduced the inhibitory activity of TBA. Substitution of G8 of the central TGT loop had a minor effect as demonstrated by the 2-fold increase in the inhibition constant. Later, c^7^G was also incorporated into the DNA sequences forming tetramolecular architectures, such as the TG_4_T and TG_5_T [222]. The modified oligonucleotides formed the same type of parallel structures as the unmodified ones did although the Hoogsteen-type H-bonding could not be formed in the absence of the H-acceptor of N7. Due to the steric requirements of the hydrogen atom of C7, the c^7^G-tetrads became deformed as compared to the full-G tetrads, resulting in changes of stacking. This led to large reduction in the thermal stability of [TG_5_T]_4_ by the c^7^G-analogs. In general, intramolecular GQs cannot form or are severely destabilized by a single c^7^G in the core, which made c^7^G a “principle-proving” analog [229–232]. The c^7^G derivative 8-aza-7-deaza-*iso*guanine (n^8^c^7^iG) (Figure 13,** 3**) nucleotide-containing DNAs can form tetrads and also pentads and thus GQ and pentaplex scaffolds. Seela and Kröschel [39] described that the T_4_($\underline{n^{8}c^{7}iG}$)_4_T_2_ molecules self-assembled into GQs in the presence of Na^+^ or Rb^+^, whereas they formed pentaplexes with Cs^+^ cations. Structural stability of the GQ assemblies is not known.

*Pyrene-Perylene dA*. GQ-induced FRET (fluorescence resonance energy transfer) has been studied by substituting loop adenosines at position 8 with a tethered FRET pair in A(htel-21), A(G)_n_TT**Py**A(G)_n_TT**PerA**(G)_n_TTA(G)_n_, where the* n* changed from 2 to 4, Py is pyrene (donor), and Per is perylene (acceptor) group (Figure 13,** 4**,** 5**). The substituted bases did not hinder the formation of intramolecular 2-, 3-, and 4-tetrad GQs in K^+^; however, they marginally destabilized them by 1-2°C, relative to the unmodified control GQs [233].

*6-Methyl-isoxanthopterin*. A fluorescent G analog 6-methyl-isoxanthopterin (6MI, Figure 13,** 6**) has been incorporated into the central TGT loop (position G10) and also at the middle G-tetrad (position G13) of the GQ-forming G_3_T_2_G_3_T**G**TG**G**GT_2_G_3_ [234], whose DNA is part of the promoter region of c-MYC oncogene. The loop modification slightly decreased the thermal stability of wild-type GQ, while the tetrad modification significantly did the same. CD spectrum of the wild-type and the modified ones displayed a strong positive band at 263 nm and a minor peak at 298 nm, which the authors, probably mistakenly, called antiparallel basket and/or chair topologies. The fluorescence intensity of the loop-modified GQ was greater than the tetrad substituted, as the authors expected from the extent of stacking interaction, which is larger in the G(6MI)G sequence than in the T(6MI)T sequence. The fluorescence emission intensity decreased when 6MI was incorporated into single-stranded oligonucleotide and decreased further in the double-stranded oligonucleotide, demonstrating that its fluorescence emission intensity can be used to probe the microenvironment of 6MI. The 6-methyl-isoxanthopterin was also substituted for the T4 (loop) of (G_3_T)_3_G_3_ oligonucleotide to develop fluorescent detection methods. The wild-type sequence folded into parallel GQ in K^+^. The substitution did not change the original topology but destabilized the GQs. *T*_*m*_ of the (G_3_T)_3_G_3_ GQ, measured in 10 mM KCl, was 90°C, which was reduced by 12°C by the base analog [124].

*2-Pyrimidinone and 4-Thiouracil Nucleosides*. Mammalian telomeric DNA is transcribed into RNA that contains the 6-mer uuaggg repeats [235]. Sequence analogs of this, like the uagggu, and those containing analogs of uridines, the zebularine (ribonucleoside of 2-pyrimidinone) that does not have the O4 atom, and the 4-thiouridine (s^4^U) (Figure 13,** 7**,** 8**), in which the sulfur atom cannot develop H-bond, have been prepared. The tetramolecular parallel GQs formed by the wild types have been radically destabilized by s^4^U: *T*_*m*_ value of 79°C for [uagggu]_4_ was reduced in [uaggg$\underline{s^{4}U}$]_4_ to 49.4°C [236]. Mendelboum and coworkers [237] prepared 4-thiouracil-2′-deoxyuridine- (s^4^dU-) containing TBA oligonucleotides, in which the base analog substituted for thymines of the loop sequences. Replacement of four thymines by s^4^dU in the 15-mer resulted in G_2_$\underline{s^{4}U}$TG_2_$\underline{s^{4}U}$G$\underline{s^{4}U}$G_2_T$\underline{s^{4}U}$G_2_. The TBA analog showed increased anticoagulant and antithrombotic properties relative to the unmodified TBA. The increased activity was explained by the altered properties of s^4^dU as compared to thymine. The substitutions rendered the molecule more hydrophobic, which might have been preferable for the aptamer-thrombin interaction; furthermore, the s^4^dU-containing oligonucleotides have been described as highly resistant to cellular nucleases. This aptamer analog contained more than 26% of thiolated nucleotides, and thus it must have been more stable in a biological environment than its unmodified counterparts. Thermodynamic stability of the s^4^U-TBA GQ was not specified [237].

### 4.2. Destabilizing Substitutions of the Sugar and Backbone Moieties

**Figure 14:**
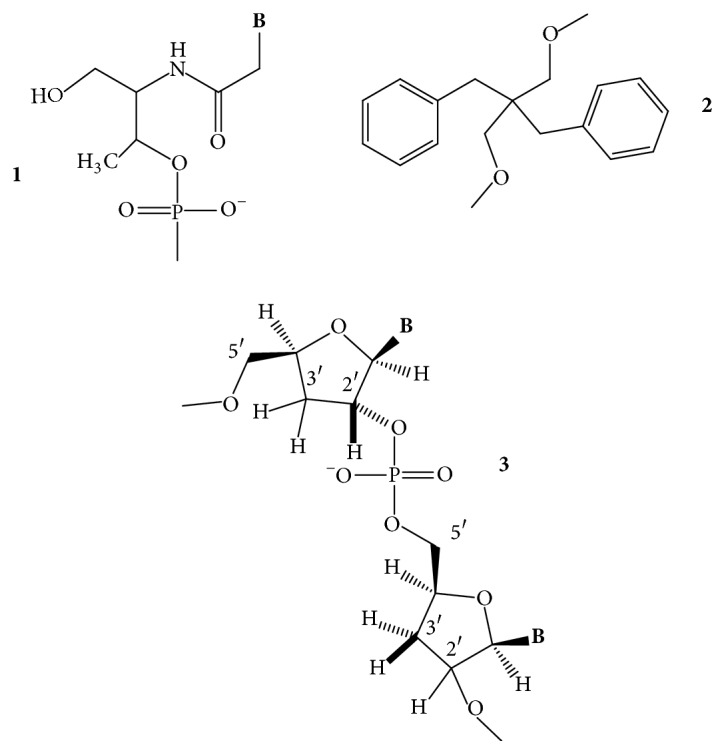
The acyclic threoninol (aTNA) nucleoside (**1**); the dibenzyl linker (**2**); and a 3′-deoxy-2′-5′-*iso*-dinucleotide (**3**).

*Acyclic Threoninol*. An acyclic sugar analog, the acyclic threoninol (aTNA)-guanine (Figure 14,** 1**), has been substituted by Zhou's group [238] for each guanine, one by one in TG_4_T. Based on the CD spectra, all the modified oligonucleotides could form GQ structures and only the G3-substituted TGG**G**GT's conformation differed from the tetramolecular parallel GQ structures formed by the natural counterpart, [TG_4_T]_4_. This modified oligonucleotide built multiple scaffolds. Thermal stability of [TG_4_T]_4_ was reduced by the aTNA-G in each case, and the modification at the 5′- and 3′-terminal G-tetrads was the most detrimental to the stability [238].

*Dibenzyl Linker*. TBA is primarily known as an anticoagulant aptamer. Another less known biological activity of TBA is its anticancer potential, which is rather hindered by the anticoagulant action, as reported by Scuotto et al. [239]. They found that replacing one residue of the TT or TGT loops with a dibenzyl linker (Figure 14,** 2**), with which seven new GQ-forming TBA sequences were created, could maintain the antiproliferative activity over the anticoagulant activity. Most T-substitutions only slightly affected the thermal stability of the wild-type TBA, except the T9-modification that reduced *T*_*m*_ of TBA (50.7°C in 90 mM KCl) by 15°C. The T13-modified analog possessed selective antiproliferative activity, while the T12 analog retained the potent anticoagulant activity of the unmodified TBA. Structural analyses indicated that the different localization of the two benzene rings of the linker was responsible for the loss of the antithrombin activity of the T13 analog.

*Methylphosphonate*. In the methylphosphonate (P-Me, Figure 12,** 3**) internucleotide linkages, a methyl group replaces one of the nonbonded oxygen atoms, and thus the internucleotide linkage becomes uncharged, neutral. This causes the ion-solvating water spine-perturbed along the backbone, resulting in the destabilization of a folded structure. With P-Me linkages-containing [TG_4_T]_4_, [G_4_T_4_G_4_]_2_, and the TBA GQs, no thermal transition was detected, and the AG_3_(TTAG_3_)_3_ GQ was powerfully destabilized [1].

*isoDNA*. The name refers to the modification of the internucleotide phosphodiester linkage, in which the natural phosphodiester bond, 3′O-OP(O^−^)-5′O, of nucleic acids is changed, for instance, to 2′O-OP(O^−^)-5′O, which is the 2′-5′*iso*DNA (Figure 14,** 3**). The fully 2′-5′ linked* iso*TBAs formed unimolecular antiparallel GQs in the presence of K^+^ ions, similar to how the unmodified, 3′-5′-linked TBA oligonucleotides did. The isomeric TBA had lower thermal stability than the native one. The TBA's *T*_*m*_ value in K^+^ was 52°C, while the* iso*TBA GQ's was 37.1°C. When the T7 and T9 were replaced by U nucleotides, the stability increased up to 45°C. On the other hand, the* iso*TBAs exhibited higher stability against exonucleases and were capable of retaining the biological function of the native TBA, that is, slowing down the process of blood clotting [240]. The antiparallel chair-type TBA GQ, whose nucleotides are connected via 3′-5′ phosphodiester linkages, folded into parallel GQ when the 2-3-2-nucleotide-long loops (TT…TGT…TT) were shortened. The 2′-5′-*iso*TBA analog, interestingly, retained the antiparallel topology with the shorter loops such as 2-2-2 or 1-3-1 or even 1-1-1 nucleotides of the resulting 14-, 13-, or 11-mer GQs. Thermal stabilities of the GQs were, however, reduced by the introduction of shorter loops into the 2′-5′-*iso*TBA sequences, from *T*_*m*_ of 48°C for the unmodified 3′-5′ TBA to 34°C for the* iso*TBA(232) and down to 20°C for the* iso*TBA(111) in 100 mM KCl [241].

## 5. Natural Base Lesions and Epigenetic Modifications in GQ DNAs

Exogenous and endogenous chemicals and radiation cause many types of lesions in cellular DNA. The nucleotides are modified by different types of reactions, such as oxidation, alkylation, and hydrolysis. These alterations are widespread and play an important role in changing the physiological states of cells and can thus lead to various diseases. Majority of genetic impairments are believed to originate from oxidative processes, which are the basis of mutation, aging, cell death, and carcinogenesis [242–244]. Reactive oxygen and nitrogen species (ROS and RNS) arising endogenously and originating mainly from the cell's aerobic metabolism also contribute to the age-dependent diseases [244–246]. There are ~80 known DNA defects that can form upon the initial attack of ROS and RNS [247]. Among the exogenous effects, the damage can arise from radiation, directly from the ionizing energy or indirectly from hydroxyl radicals that form by the ionization of the solvation shell around the DNA [248, 249]. The radical cation (electron ionization hole) can travel hundreds of Angstroms by hopping before being entrapped, preferentially by purine bases [250, 251]. Oxidation of purines initially leads to 8-oxo-7,8-dihydroguanosine (o^8^G), 8-oxo-7,8-dihydroadenosine (o^8^A), and 8-oxo-7,8-dihydroinosine (o^8^I) in RNA, in DNA, and in the nucleotide pool [244]. The effects of natural base lesions on the structure and stability of double-stranded DNA models have been extensively studied (see [252–258] and the references therein). Majority of the lesions are promutagenic and procarcinogenic if not repaired in due time. The base excision repair (BER) pathway, regulated by many different types of enzymes including DNA glycosylases, abasic endonucleases, phosphodiesterases, DNA polymerases, and DNA ligases, is responsible for the accurate removal of the lesion and the restoring of the original state of the double-stranded nucleic acids [243]. Due to imperfections of the repair system, the lesions that are not repaired can severely damage the DNA structure where they are formed. Base damage also occurs in noncanonical DNA structures such as the GQs. Studying the lesion-damaged GQ structures using various GQ models is less than a decade old. A few cases are known only about repairing damaged GQs, such as the N6-methylguanine (m^6^G) [259] and the further oxidized derivatives of 8-oxoguanine (o^8^G), but o^8^G is not among them [260].

Using GQ models, the o^8^G, o^8^A, n^8^A (8-aminoadenine), m^6^G, G- and A-abasic (AP) sites, hm^5^U, hypoxanthine (I), xanthine (X), and the cyclobutane thymine dimers have been investigated for the effect on GQ stability. Most natural DNA damage is destabilizing both in canonical and in noncanonical nucleic acids; a few, however, do not affect or even stabilize the GQ fold. The stabilizing lesions are the o^8^A and n^8^A, only if located in the loops of the htel GQs, and also o^8^G if located in the tetramolecular DNA GQs of [TG_4_T]_4_ and [TG_5_T]_4_. hm^5^U, with its minor effects, also belongs to this group of analogs as well as the AP site when replacing a single-base loop in GQs of the T(G_3_T)_4_ and (G_2_AP)_3_G_2_.

### 5.1. Natural Base Lesions That Stabilize or Destabilize, Depending on the GQ Structure

**Figure 15:**
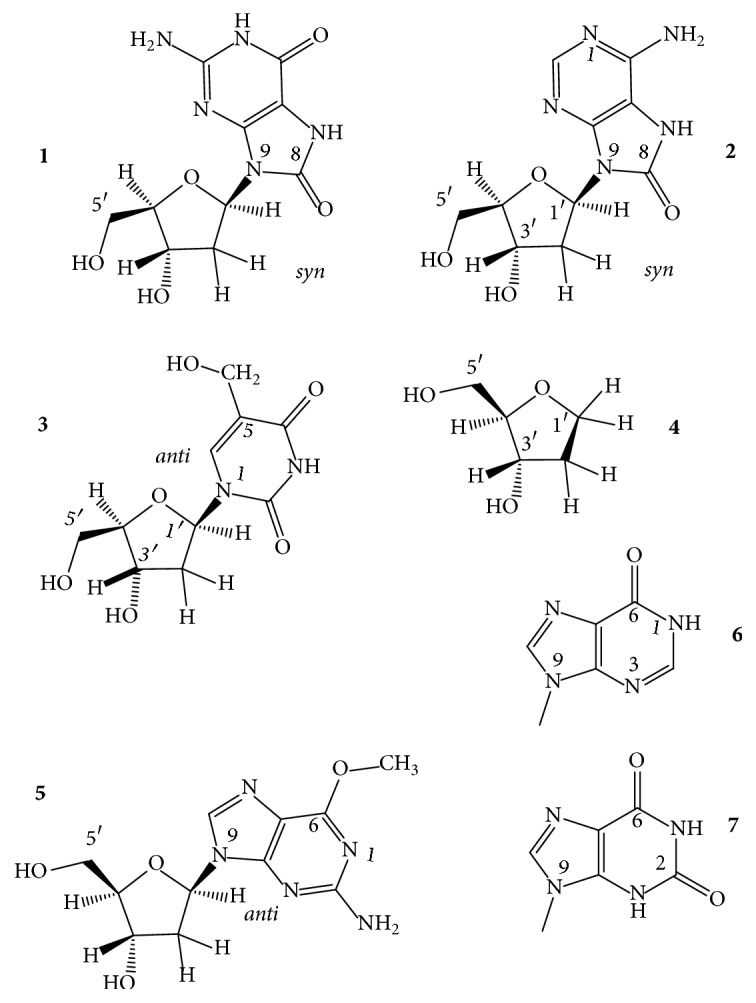
Structures of the* syn* 8-oxo-2′-deoxyguanosine (**1**);* syn* 8-oxo-2′-deoxyadenosine (**2**);* anti* 5-hydroxymethyl-2′-deoxyuridine (**3**); the tetrahydrofuranyl abasic site (**4**);* anti* O6-methyl-2′-deoxyguanosine (**5**); hypoxanthine (**6**); and xanthine (**7**).

*8-Oxo-*2′*-deoxyguanine (o*^8^*dG)*. Guanine has the lowest redox potential among the four DNA bases [251]; therefore, guanine is the major site of oxidation in DNA by ROS, the reactive oxygen species [261]. Among the numerous oxidative derivatives, the 7,8-dihydro-8-oxoguanine (8-oxoguanine, o^8^G, Figure 15,** 1**) was found to be the major primary product* in vivo* [262–264]. Since* in vivo* levels of o^8^G were found to be safely measurable, being between 0.3 and 4 o^8^G per 10^6^ G, o^8^G has been used as a marker of oxidative stress of cells [265, 266]. o^8^G is even more prone to oxidation than G as its redox potential is even lower [267]. Further oxidation of o^8^G results in guanidinohydantoin and spiroiminodihydantoin, among other minor products [268, 269]. Consecutive runs of guanines, as those in the potential GQ-forming G-rich sequences, further lower the redox potential of guanine since these runs act as sinks for the oxidative damage [270]. The glycosylases known to repair o^8^G in duplex DNA, such as OGG1, NEIL1, and NEIL3, cannot remove o^8^G from DNA GQs, although these enzymes do repair guanidinohydantoin and spiroiminodihydantoin in GQs [260]. Therefore, the unrepaired, persistent o^8^Gs can ruin the GQ structure. This has been suggested to lead to telomere shortening and finally to cellular senescence [271]. The effect of o^8^G was, however, recently proved by Fouquerel and coworkers to be not unambiguous, as the destabilized, partially unfolded GQ could promote telomerase activity that could lead to telomere extension [272, 273]. Formation of o^8^G in GQs motivated wide-ranging investigations.

The 8-oxoguanine was first studied with tetramolecular parallel GQ structures by Gros et al. [222] using the [TG_4_T]_4_ and [TG_5_T]_4_ models. These GQs assemble from four TG_4_T and TG_5_T strands, respectively, and therefore the presence of a single modified base in one strand forms a modified tetrad in the GQ. Incorporation of a single o^8^dG into each G-position of TG_4_T and TG_5_T did not hinder the formation of tetramolecular parallel GQs. Interestingly, o^8^dG proved to be a stabilizing modification, especially when incorporated at the 5′ position of the G4 or G5 sequence, as characterized by the* T*_1/2_ values. As the preferred glycosidic conformation of o^8^dG is* syn* (Figure 15,** 1**), similar to the majority of 8-modifications of G [63], and in most cases in the parallel GQs all nucleosides have* anti* glycosidic torsion angle, o^8^G was supposed to destabilize the structure. Furthermore, in the 6,8-diketo tautomeric form of o^8^G [274], the donor-acceptor arrangement of the Hoogsteen H-bonding changes, which again would have predicted the destabilization of the parallel GQ by the o^8^dG tetrad. The observed stabilization effect was finally explained by strong stacking interactions with the neighboring* anti* G-quartet. As it turned out later, the elevated stability was observed merely with these two tetramolecular scaffolds. There was another experiment for tetrad formation from o^8^G nucleotides when o^8^dG replaced G in the four 5′ positions of the GGG triplets (in bold, underlined) in **G**GGT**G**GGT**G**GGT**G**GG and results were the opposite, destabilization. This oligodeoxynucleotide folded into an intramolecular parallel GQ, possibly due to the single-base loops. Thermostability of wild type was substantially reduced by o^8^dG, by 10°C in Na^+^, and no unambiguous *T*_*m*_ value could be determined in K^+^ where an even larger destabilization was apparent. The circular double H-bonded scheme was assumed to consist of N1H-O8G and O6G-HN7 [275].

Reduced stability and conformational changes were observed when a single o^8^dG was incorporated into the GQ-forming htel oligonucleotides. Szalai et al. [276] used the 25-mer model A(htel-21)TGT and found that o^8^G at 5′ positions of the GGG triplets retained the intramolecular antiparallel topology of the wild type, whereas o^8^G in the middle positions of the triplet caused formation of multiple folds. With the 24-mer hybrid GQ of TT(GGGTTA)_3_GGGA, Lech et al. [80] detected significant destabilization of the wild type even in cases when the* syn *dGs were changed to o^8^dGs in positions 3, 9, and 15 (all 5′ positions in the GGG triplets) and the Δ*T*_*m*_ values ranged between −9.8 and −18.5°C. Concurrently, a mixture of topologies was formed. o^8^dG was also incorporated into all 12 dG positions (*syn* and* anti*) one by one, into the GQ-forming htel-21, G_3_(TTAG_3_)_3_ by Sagi and coworkers [26]. The study revealed that the GQ structures were substantially destabilized by enthalpy-driven effects both in Na^+^ and in K^+^ solutions. The negative effect was position-dependent and varied with the cation used. When the single o^8^dG was located in the two terminal tetrads, the Na^+^-stabilized basket-type antiparallel topology was also retained in K^+^, contrary to the wild-type htel-21, which forms a mixture of folds, hybrids, and K^+^-antiparallels in K^+^ [68, 101, 277, 278]. The middle tetrad substitutions by o^8^dG caused the largest reductions in stability (−19 to −26°C in Na^+^ and −27 to −30°C in K^+^), as observed before with other base mutations [279, 280], and various folds were observed too. The damaging effect of o^8^dG on the stability of monomolecular htel GQs was surprising in light of its stabilizing effects with tetramolecular GQs [222] (the o^8^dG triggered only marginal destabilizations in deoxyoligonucleotide duplexes and the effect was also length-dependent: with duplexes of 15 nucleotides long, even a small stabilization was observed; see references in [26]). In an o^8^G-G-G-G tetrad, the 8-substitution changes the Hoogsteen-type circular H-bonding pattern. In the 6,8-diketo tautomeric form of o^8^G [274], the N7 is a hydrogen donor, instead of acceptor, and therefore only a single H-bonding pattern remains when o^8^G is present in the tetrad. The weakened H-bonding and the stacking changes induced by the altered tetrad can result in the extensive destabilization of the GQ.

As o^8^dG contained in htel GQ was found not to be a substrate of the glycosylase enzymes that repair o^8^dG in duplex DNA, such as the hOOG1 and NEIL enzymes [260], o^8^G can become a persistent lesion in GQs. Surprisingly, this persistence was without any immediate negative consequences on the stability of telomere complexes* in vivo* (see references in [281]). An and coworkers have built a 31-mer telomeric GQ model from TAGGG(TTAGGG)_4_TT, which contained five G-triplets, instead of the usual four, in which the dGs at the 5′-positions of 3, 15, and 27 were replaced by o^8^dG. These modified oligodeoxynucleotides folded into hybrid-1 or hybrid-2 GQ form. They observed that the folded structures could effectively accommodate a single o^8^dG by looping out the damaged G-tract and allowing the other four G-triplets to adopt the hybrid fold. This change caused only a minimal negative impact on the stability of the GQ [281], contrary to the results obtained with the four G-tract telomeric GQ models* in vitro*. Considering the availability of additional G-tracts in the telomeric complexes, the authors explained how the persistent presence of a lesion can exist without immediate destabilization effect on the DNA-protein complex* in vivo*.

Another way was also found to offset the intense destabilization caused by a single o^8^dG of the GQs built from the four TTAGGG repeat htel sequences and this was by the proper site-specific incorporation of another modified base, specifically xanthine (2,6-dioxopurine, X, Figure 15,** 7**). Xanthine is another natural derivative of guanine (see in the section Xanthine). X can also be assumed to be a destabilizing analog due to the loss of H-bonds if incorporated into a G-tetrad, similarly, as it was found with hypoxanthine (I for inosine) (see under section Hypoxanthine). The outcome of the o^8^dG-X double modification depends on the positions of the modified nucleotides. Benz and Hartig [282] incorporated first two o^8^G and two X nucleotides into the TA(htel-21)T sequences, which folded into X:o^8^G:X:o^8^G tetrads-containing GQs. The modified tetrads destabilized the wild-type GQ and the topology depended on the arrangement. The GQ either remained in the basket-type antiparallel or changed into parallel form:

- wild-type: TA GGG TTAGGG TTAGGG TTAGGG T *antiparallel*
- o^8^G(3):X(11):X(15):o^8^G(23): TA $\underline{O}$GG TTAGGX TTAXGG TTAGGO T* antiparallel*
- o^8^G(3):X(9):o^8^G(15):X(21): TA $\underline{O}$GG TTAXGG TTAOGG TTAXGG T* parallel*

Later, Cheong et al. [283] found the positions for o^8^G and X that could compensate for the negative effect of o^8^G on the stability of the wild type and which also retained the original topology. They inserted the G:G:X:o^8^G tetrads into a series of TT(htel-21)A deoxyoligonucleotides. Conformation of the modified GQ scaffolds remained similar to the original hybrid-1 fold. Three of the modified GQs were destabilized, by 3°C to 12°C in their *T*_*m*_; two had similar stability as the wild type had (56°C), and the G(3):o^8^G(9):X(17):G(21) tetrad proved to be stabilizing, by 2°C in its *T*_*m*_ relative to the wild type. Cheong and coworkers [284] could also reverse the polarity of H-bonds in a tetrad while the original folding topology of the wild type was preserved.

The effect of o^8^dG has been well demonstrated to depend on the secondary structure of nucleic acids [26, 285, 286]. Recent studies provided additional results, such as the comparison of the effect of o^8^dG in triplexes and GQs [100], the involvement of o^8^dG in the stabilization of “guanine-vacancy-bearing” GQs [287], or a comprehensive study on the complex effect of metal ions and cosolutes on the topology of the A(htel-21) GQ when both o^8^G and xanthine were contained in the tetrads [288]. 8-Oxoguanine in GQ loops accommodated well and did not have much effect on the stability [281]. The Zn(II)-porphyrin complex-induced oxidation of guanines in the GQ of TA(htel-21) yielded o^8^G and its further oxidized product, the spiroiminodihydantoin. The oxidized bases prompted structural rearrangements of the parallel and hybrid TA(htel-21) GQs into an antiparallel-like conformation [289], such as what was observed earlier with o^8^G-containing htel-21 GQs [26].

*8-Oxo-*2′*-deoxyadenosine (o*^8^*dA)*. The first study with 7,8-dihydro-8-oxo-2′-deoxyadenosine (8-oxodA or o^8^dA, Figure 15,** 2**), another oxidative natural base lesion, was carried out by Esposito et al. [290] and Petraccone et al. [291] using the tetramolecular parallel GQ DNA models of [AGGGT]_4_ and [TAGGGT]_4_. All the modified oligonucleotides formed the same parallel-type tetramolecular GQs as the unmodified two oligonucleotides did. The o^8^dA substituting for dA nucleotides in both structures decreased the thermostability of the parent GQs by 14°C and 8°C, respectively, according to absorption-based *T*_*m*_ measurement, and the drastic negative effects were supposed to originate from the formation of the o^8^dA-tetrads [290]. Calorimetry, however, provided different results: the [AGGGT]_4_ GQ was not destabilized by the analog, formulated as 8-hydroxy-dA, oh^8^dA [291].

Among the htel GQ models, o^8^dA was first incorporated into the A(htel-21) GQ by Aggrawal et al. [292]. The analog moderately stabilized, did not affect, or slightly destabilized the GQ depending on the position of substitution. Single, double, and triple substitutions by o^8^dA were also investigated. In Na^+^ the average Δ*T*_*m*_ of four single substitutions was ~0.5°C above the wild-type's *T*_*m*_ of 60.1°C, 1°C in Δ*T*_*m*_ of three double modifications, and 4.1°C of the triple. In 110 mM K^+^ solution the respective values were 2.5°C, 2.7°C, and 8.9°C. The presence of o^8^A in the loops of A(htel-21) GQ did not change the intramolecular antiparallel conformation in Na^+^, but in K^+^ multiple folds were shown by the CD spectra. The stabilization by o^8^A was explained by the tight binding of K^+^ into the pocket formed by the O8 of o^8^A and its loop [292]. In another study [293], the effect of o^8^A was compared with the effect of other natural loop lesions, such as the adenine abasic (A/AP) site and 5-hydroxymethyluracil (hm^5^U) in the GQ of A(htel-21). o^8^A stabilized the Na^+^-basket and the K^+^-stabilized folds by 1.7°C and 1.1°C, respectively, as averages of the effects at the four sites. The hm^5^U (Figure 15,** 3**) hardly affected the stability, while the AP site (dSpacer; Figure 15,** 4**) destabilized the GQ structure [293].

*5-Hydroxymethyluracil (hm* ^5^ *U)*. One of the main natural oxidation products by ROS of the DNA thymine is hm^5^U (Figure 15,** 3**). The ten eleven translocation (Tet) enzymes oxidize the epigenetically important base 5-methylcytosine stepwise to 5-hydroxymethylcytosine, 5-formylcytosine, and 5-carboxycytosine. Pfaffeneder et al. [294] found that Tet enzymes also oxidized T to hm^5^U in mouse embryonic stem cells. The hm^5^U also forms by ionizing radiation and oxidation of the deaminated m^5^C [295]. hm^5^U was first incorporated into the 5′-T of**T**GGGT oligonucleotide that readily formed the tetramolecular parallel GQ. hm^5^U was modeled as forming an hm^5^U-tetrad via an extra H-bond through the 5-OH group [296]. Virgilio et al. [297] replaced each thymine in the three loops of the TBA DNA sequences by hm^5^U. All six sequence analogs retained the ability of the native TBA oligonucleotide to fold into the antiparallel, chair-like, two-tetrad GQ. hm^5^U did not affect or slightly, by 1–3°C, increased the thermal stability of the native GQ, except for the hm^5^U in position 9 of the central TGT loop, which increased *T*_*m*_ of the native by 6°C. All TBA analogs showed decreased affinities to thrombin [297]. Effect of hm^5^U on the stability and conformation of htel GQs was described in 2015 by two laboratories [293, 298]. Both groups found that the replacement of a T of a TTA loop by hm^5^U resulted only in negligible effects on stability and did not affect the intramolecular topology of the native GQ. Sagi and collaborators [293] replaced each of the six T nucleotides in A(htel-21), the AGGG(TTAGGG)_3_ and the Δ*T*_*m*_ values, determined by absorption-based thermal melting profiles ranged from −1.2°C to 0.5°C in Na^+^ and from −0.5°C to 0.8°C in K^+^ solutions, both containing 0.169 mM of the cations. Virgilio et al. [298] observed only stabilization by hm^5^U when six thymines were substituted either in A(htel-21) or in the 26-mer (TTAGGG)_4_TT DNA GQs. With the former GQ, the Δ*T*_*m*_ values ranged between 0.5 and 2.4°C and with the latter between 0.2 and 2.4°C. *T*_*m*_ values were determined here by calorimetry in 100 mM K^+^ solutions.

*Abasic Site (AP Site)*. Purine and pyrimidine abasic sites (AP sites) are among the most frequent lesions in cellular nucleic acids formed via spontaneous base loss, mainly depurination, and as intermediates in the enzymatic base excision repair process of various other base lesions [299]. Thousands of purine bases are released from DNA in every human cell daily and the resulting AP sites are highly mutagenic if they are not repaired. Natural AP sites occur in equilibrium states between the hemiacetal and the aldehyde forms. Due to the aldehydes, the AP sites are unstable leading to DNA chain breaks through beta-elimination [299]. Therefore, mostly stabilized forms of AP sites, primarily the tetrahydrofuranyl analog (dSpacer, Figure 15,** 4**), have been used in studies of abasics with canonical, double-stranded oligodeoxynucleotide models (e.g., [252, 253]). With noncanonical models, the effect of abasic lesions was a rather unexplored area until up to a decade ago [20, 300, 301]. With GQs, the well-known tetramolecular model assembling from TG_5_T was first studied in 2010 by Esposito et al. by replacing the dGs by dSpacer AP sites (G/AP) [302]. In the same year, a natural model, the GQ of a human telomeric repeat sequence G_3_(TTAGGG)_3_, the htel-21 was investigated for the effect of loss of G in G-tetrads by Sagi and coworkers [303]. Later, the GQ of the A(htel-21) was used as a model by Fujimoto's group [304]. This study also examined the effect of the crowding environment on abasic scaffolds. In 2012, the TA(htel-21) GQ was the abasic model of Virgilio et al. [305]. The main and shared conclusions of these studies were that the presence of a single G/AP in the G-tetrads did not hinder the formation of the tetramolecular or the monomolecular scaffolds. On the other hand, a single G/AP significantly decreased the thermal and thermodynamic stability of the parent GQ in a position-dependent way. With the three-tetrad htel scaffolds, the substitutions in the middle tetrad led to the largest reduction of stability. For instance, in K^+^ a single G/AP incorporated into the two outer G-tetrads of the three-tetrad GQ of htel-21 decreased *T*_*m*_ of 73°C (Δ*G*°_37_ −6.44 kcal/mol) of the wild type by 10–17°C and by 1.76–3.43 kcal/mol in free energy changes (ΔΔ*G*°_37_) and by 21–26°C in the middle tetrad. (Due to the formations of multiple folds in the middle tetrad-substituted sequences, no thermodynamic data were calculated from the absorption-temperature profiles.) In Na^+^ the wild-type GQ (*T*_*m*_ 68°C, Δ*G*°_37_  −4.75 kcal/mol) was destabilized by 3–17°C and 0.56–2.92 kcal/mol in the outer tetrads and by 12–23°C and 1.99–3.8 kcal/mol in the middle tetrad [303].

The substitution of the loop-T4 sequence of G_4_T_4_G_4_ by AP sites [306] did not prevent the oligonucleotides from assembling into a dimeric, fold-back GQ either in Na^+^ or in K^+^, although thermal stability of the unmodified GQ was found to be reduced. With the TBA GQ built by the GG**T**TGG**T**G**T**GG**T**TGG, in which the bold-underlined Ts were substituted with an AP site, one by one, the AP site in T9 considerably destabilized the native TBA GQ, whereas, the AP sites in the other positions moderately stabilized the unmodified TBA [307]. Depurination of a TTA loop (A/AP) of A(htel-21) also reduced the structural stability of the native GQ. The Δ*T*_*m*_ values ranged from −4.0 to −6.7°C in Na^+^ and from −0.2 to −6.5°C in K^+^ [293, 308]. These were much less drastic effects than those caused by the G/AP sites in the tetrads of the same GQ model [303]. Folding topology was, however, changed by the loop A/AP sites in a position-dependent way [293, 308]. The wild-type A(htel-21) GQ is polymorphic in K^+^ consisting of hybrid-1 and hybrid-2 as well as K^+^-stabilized antiparallel structures [309]. The loss of a guanine in any G-tetrad shifted the equilibrium towards the antiparallel type [303], whereas, with two loop A/AP abasic GQs (A/AP in the first loop, abbreviated as ap7, and in third loop, ap19), a shift towards the formation of higher population of parallel strands in the mixture was observed. This means the shifting of conformational equilibrium towards higher concentrations of the hybrid folds. Actually, ap19 turned out to be a pure hybrid-2 type fold and the ap7 a hybrid-1 architecture, although the latter formed clearly only at high, millimolar strand concentrations. Folding topology of the wild-type htel-21 and A(htel-21) GQs has been found to depend on the oligonucleotide strand concentrations and to change qualitatively above 2 mM of strands [310–313]. Polymorphism increased when the A/AP was located in the middle loop at position 13, and this was the ap13 structure(s). The A/AP site in the GQs of ap7 and ap19 was located in propeller-like loops. The abasic GQs transformed easier than the wild type into parallel GQs under dehydrating conditions (57% ethanol). The GQ of A(htel-21), in which a loop adenine was replaced by an AP site, the ap7, ap13, and ap19, folded into parallel GQ in K^+^ even in the absence of ethanol. This study [308] showed that the loop adenine AP sites are potent tools to fine-tune the folding arrangements of the telomeric GQs. The loop-abasic site-induced “monomorphism” has been confirmed by Wu et al. [314], who used this result for a sensor platform. They stated that an AP site replacing a loop adenine can extremely narrow the structure distribution to a specifically monomorphic GQ conformer, depending on the position of the AP site.

Loop thymidines have also been changed to AP sites. Rachwal and coworkers. [129] replaced the loop thymidines in the propeller-type single-base loops of the monomolecular parallel GQ assembled from the 17-mer T(G_3_T)_4_. The substitution did not change the conformation; however, surprisingly, it increased the thermostability of the structure [129]. Another study by Esposito et al. [315] found that single AP sites replacing loop Ts in the GQs of the 16-mer aptamer (GGGT)_4_ (T30923 or T30695) were not able to affect significantly the conformation and stability of the original structure. The original folding topology was a 5′-5′ dimer of two stacked parallel GQs, each containing three G-tetrads and three single-thymidine reversed-chain loops. This aptamer GQ has been reported to exhibit anti-HIV activity by targeting the HIV integrase. With four such GQ analogs, authors could shed light on the steric interaction of the GQ with the integrase [315]. (A former study [20] also showed that the AP site-containing TBA GQs have altered biological activity: an AP site substituting for any G residue of a G-tetrad in the TBA GQ significantly reduced the inhibition of thrombin. Substitution for either T4 or T13, which stack on the tetrad, also significantly diminished the inhibitory activity of the TBA GQ [20].) Sekridova et al. [316] studied the single-nucleotide loop-containing, two-tetrad GQ of G_2_AG_2_CG_2_AG_2_ from the human ALU-repeat fragment, which was characterized by parallel topology and high thermodynamic stability. The loop bases were replaced with the flexible, nonnucleotide triethylene-glycol (teg) or a tetrahydrofuranyl AP site. The triethylene linker in (G_2_teg)_3_G_2_ decreased *T*_*m*_ of the native GQ significantly, from 74°C (in 100 mM KCl) to 50°C, while the AP site in (G_2_**AP**)_3_G_2_, surprisingly, increased *T*_*m*_ considerably, up to 85°C. Furthermore, AFM (atomic force microscopy) data suggested that the two-tetrad GQ with the abasic loops was prone to dimerization in 10 mM KCl. The dimers were supposed to be stacks of monomolecular GQs. At high concentration, 200 mM, of KCl, this loop-abasic GQ formed higher-order aggregates, that is, short nanowires of ~20 nm length, that were long G4-stacks [316]. The loop-abasic sites have been used in several other GQ model studies, such as in the ribo analog of the telomeric DNA and TERRA [169] and also with A(htel-21) [313]. Heddi et al. [317] investigated the formation of GQs containing 4n-1 guanines in the core and synthesized, among others, the T_2_G**G**GT(G_3_T)_3_ oligodeoxynucleotide, where the **G** is for the G/AP site in the middle tetrad of the three-tetrad GQ. The G/AP site drastically reduced the thermal stability of the unmodified parallel GQ, down to 51.2°C from >85°C in 60 mM K^+^ [318].

### 5.2. Destabilizing Natural Base Lesions

*O6-Methylguanine (m* ^6^ *G)*. O6-Methylguanine is a major base lesion of nucleic acids* in vivo* [319], which is promutagenic and procarcinogenic [320]. The* anti* m^6^dG (Figure 15,** 5**) destabilized the tetramolecular GQ [TG_5_T]_4_ in a position-dependent manner in Na^+^ by forming m^6^dG-tetrads, which did not change the parallel characteristics of the CD spectrum of the wild-type GQ [222]. Incorporated in place of each G at the 5′-end G-triplet of AG_3_(TTAG_3_)_3_, which represented each of the three G-tetrads, the m^6^G reduced the thermal stability of the unmodified GQ. The largest destabilizing effect was observed with the middle tetrad substitution, both in Na^+^ and in K^+^. CD spectrum of the unmodified 22-mer in Na^+^ was only slightly modified by m^6^G. In K^+^, the unmodified oligonucleotide formed hybrid-type mixed folds but the spectra of the outer tetrads-modified GQs looked like their Na^+^-spectra. Spectrum of the middle tetrad-modified structure was qualitatively different from the others. The decreased thermal stability might have been the consequence of missing cation coordination, the disrupted circular H-bonding, and the reduced stacking interactions [321]. Bulky substituents linked to O6 of G, such as the O6-p-nitrobenzyl group, completely hindered the formation of GQ from the TBA oligodeoxynucleotide when substituted at G1. Adding Na_2_S_2_O_4_ to the solution of the modified sequence resulted in the formation of GQ, as the CD spectra showed, due to the removal of the reduction-sensitive p-nitrobenzyl group [322]. m^6^G is one of the few natural base lesions that was found so far to be repaired. O6-Alkylguanine-DNA alkyltransferase (AGT) is the repair enzyme that removes the promutagenic O6-alkylguanine adducts from duplex DNA* in vitro* and* in vivo*. Using m^6^G-substituted A(htel-21) GQs, Hellman et al. [259] found that AGT repaired the m^6^G adducts located within the folded GQ, and the rate of repair was comparable to those found with duplex DNAs under analogous conditions. Repair was dependent on the position of the adduct. In general, m^6^G located in the outermost (top and bottom) tetrads of a GQ stack exhibited more rapid-phase repair than did the adducts located in the inner tetrad.

*Hypoxanthine (I for Inosine)*. Enzymatic deamination of adenine nucleosides of RNA and DNA* in vivo* leads to hypoxanthine-ribosides, that is, to inosine or to 2′-deoxyinosine [299, 323]. Hypoxanthine, $\underline{I}$ (Figure 15,** 6**), is a G analog with a missing 2-amino group, and thus the Hoogsteen-type circular double H-bonded G-tetrad loses a hydrogen bond if $\underline{I}$ is formed or incorporated into a GQ. This is supposed to lead to some destabilization of the GQ. The $\underline{I}$ base was first incorporated into the* Oxytricha* telomere repeat G_4_T_4_G_4_ sequence in 1993. The G_4_T_4_G_3_I formed a diagonally looped symmetrical bimolecular antiparallel GQ with four G-tetrads and T4-loops in both Na^+^ and K^+^ [324]. Later, $\underline{I}$ and U were both inserted into the Oxy-3.5 sequence G_4_(T_4_G_4_)_3_ that resulted in G_4_TUTUG_4_T_4_G_4_UUTTG_3_I. Both the wild-type and the modified sequences formed a monomolecular GQ with a diagonal T4 loop and two modified edgewise loops. Thermodynamic data were not published [224]. Tetramolecular structures were also investigated for the effect of hypoxanthine. The $\underline{I}$-tetrads drastically destabilized the parallel GQs of [TG_4_T]_4_ and [TG_5_T]_4_ in position-dependent ways [222]. $\underline{I}$-containing tetramolecular GQs were also built from the single repeat htel sequences TTAGGIT and TTAGGGIT and analyzed by 1H NMR [325]. Numerous intramolecular GQ-forming htel sequences were also substituted with hypoxanthine. Relative stability of an $\underline{I}$-modified htel-21 GQ was different in Na^+^ and K^+^ solutions. In position-dependent ways, the Δ*T*_*m*_ values were −10°C or more negative by single substitutions and −20°C or lower by double substations in K^+^, and −6.5°C or larger on single and −14°C or larger on double substitutions in Na^+^, as determined by Risitano and Fox [326]. With the GQ of (TAG_3_)_3_TAIGG, $\underline{I}$ did not change the CD signature of the wild-type antiparallel GQ [327], and similarly, the single $\underline{I}$ in the GQ of GIGT(GGGT)_3_ did not change the parallel CD spectrum of (GGGT)_4_ but made the GQ structure more stable for NMR studies by significantly improving the spectral resolution [328, 329]. The absence of the 2-amino group facilitates the folding transition of the unstable or equilibrium structures into another fold; that is, substitution of a dG by dI could selectively disfavor particular GQ conformations. An example for this was the study that used the dI for dG substitution to change the conformational equilibrium between competing GQ folds in AG_4_AG_4_CTG_3_AG_3_C [330]. The G-to-I substitution at various G positions in the five TTAGGG repeat htel GQs resulted in the protruding of the GIG section into a long loop. Thermal stability of the unmodified htel-25 was 62°C, which the extended GIG repeats decreased significantly. *T*_*m*_ of the htel-31(I at pos.22) was 49.8°C, that of the double-modified htel-37(I22, I28) was 47.7°C, and that of the triple-modified htel-43(I22, I28, I34) was 46.5°C [81]. With another long repeat sequence, the double G-to-I substituted TAG_3_(TTAG_3_)_7_TT, it was found that one permutation of $\underline{I}$-substitutions at the 5′-end produced a minor increase in the thermodynamic stability relative to the wild type, while four other double mutations, however, decreased it, some of them significantly [331]. G-to-I substitutions were also used with GQs of A(htel-21) and A(htel-21)T [332], the VEGFA G_3_AG_3_TTG_4_TG_3_, the c-myc Pu18 AG_3_TG_4_AG_3_TG_4_, and the PIM1 G_3_CG_4_CG_5_CG_4_ sequences [333]. Guanine in the central TGT loop of the TBA GQ was replaced by adenine or hypoxanthine to study the role of G8 in the GQ stabilization. The G-to-I substitution resulted in a slight decrease in the thermal stability; the G-to-A change triggered a larger destabilization. *T*_*m*_ values were 50.8°C, 49.5°C, and 45.0°C for the wild-type, the I-modified, and the A-modified GQs, respectively [334]. Results of a recent study [335] have shown that incorporation of dI into selected positions of AS1411 GQ can elevate its biological activity. AS1411 is a 26-mer GQ-forming aptamer with antiproliferative activity originating from selective binding to nucleolin protein. The augmented activity is thought to arise from stronger binding to the nucleolin. Based simply on the increasing amplitude of the 264 nm CD peak by dI substitutions, authors concluded that dI may also stabilize the GQ [335].

*Xanthine (X)*. Xanthine (X) or 2,6-dioxopurine or 3,7-dihydropurine-2,6-dione (Figure 15,** 7**), is an analog of guanine. It is found in all human tissues and is also a natural base derivative in DNA originating from guanine by guanine deaminase or from hypoxanthine by xanthine oxidoreductase [299]. Similar to inosine, xanthine also disrupts the Hoogsteen-type circular double H-bonding structure of a G-tetrad due to the missing NH_2_ group of G, which probably leads to a destabilized GQ fold. Not counting the o^8^G-X double modifications [282, 283, 288], the X in GQ tetrads has only been studied via in silico methods [336, 337]. A complex quantum chemical approach showed a high degree of structural and energetic compatibility between the guanine and xanthine-based GQ models. The calculations established that hydrogen bonding made the greatest (~50%) contribution to the internal stability of the DNA GQs. Base stacking and ion coordination terms proved commensurable and accounted for the rest of the energetic contributions. Using two- or three-tetrad model systems, the guanine tetrad structures benefited from the high degree of H-bond cooperativity while the xanthine tetrad models were characterized by the more favorable stacking interactions [338]. Experimental proofs for these conclusions are not yet available.

**Figure 16:**
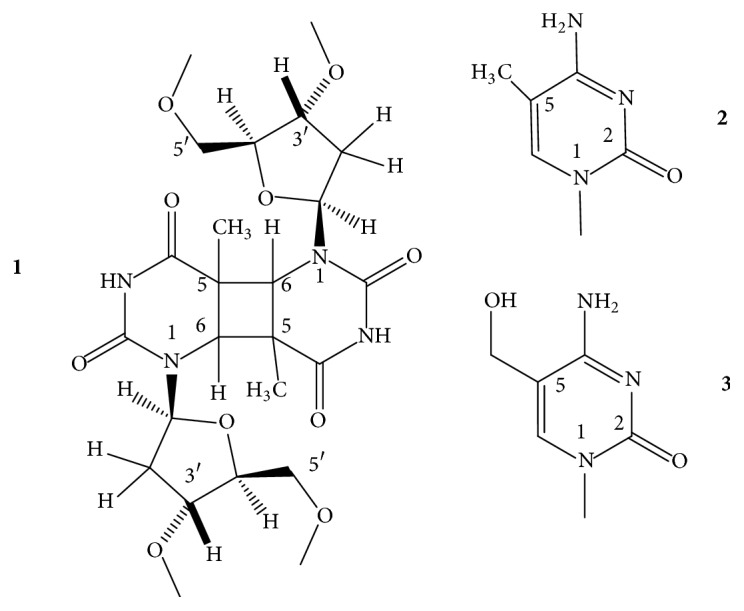
Structure of the* anti* cyclobutane thymine dimer (**1**), 5-methylcytosine (**2**), and the 5-hydroxymethylcytosine (**3**).

*Cyclobutane Thymine Dimers*. Ultraviolet B (UVB) light irradiation induces the formation of cyclobutane pyrimidine dimers in nucleic acids* in vivo*. Under physiologically relevant solution conditions, the UVB light can prompt the formation of* anti* cyclobutane thymine dimers (Figure 16,** 1**) in GQs, as Smith et al. recently reported [339]. The* anti* refers to the position of the 5-methyl groups of the two thymines of the cyclobutane ring. Stereochemistry and yield of formation greatly depended on the topology of the GQ, the cations present, and the temperature of the solution. The two-tetrad basket-type model formed from various mutated analogs of the 26-mer A_3_(htel-21)A_2_, in which two-tetrad fold was first described for the (htel-21)T in K^+^ by Phan's group [77], was found as the first GQ structure in which the thymine dimer could form in K^+^, but not in Na^+^. The T-dimer formed between the two edgewise loops. In this fold, the loop thymines are close enough for the UVB-induced cyclization reaction to occur. Other dynamic K^+^-topologies were also proposed as appropriate stereo structures for T-dimer formation. Thermal stability and conformational changes caused by the dimer formation were discussed by the authors [339].

*Clustered Lesions in GQs*. Ionizing radiation produces clustered lesions in the genetic material that results in distorted secondary structures in the double-stranded DNA. Clustered lesions have been found to be more difficult to repair, which were thus more harmful than the single, isolated ones and were detrimental to cells [340]. By definition, clustered DNA lesions form when two or more forms of damage appear in the double-stranded DNA strands within one to two helical turns, which involves 10–20 base pairs [341]. This type of lesions can also form in GQs. Sagi and coworkers [342] described for the first time the effect of clustered lesions on GQ structures, specifically clustered loop adenine (A/AP), tetrad guanine (G/AP), and A/AP + G/AP abasic sites (AP sites are among the most frequent natural lesions [299]). We have reformulated the definition described for duplex DNAs to be suitable for GQs as well: two or more lesions within a four-stranded, two- or three-tetrad core GQ unit consisting of up to 21 nucleotides in the core. For the experiments, the potential GQ-forming A(htel-21) and TA(htel-21) TT oligodeoxynucleotides were synthesized with 36 permutations of double and triple tetrahydrofuranyl AP sites. In 100 mM K^+^ solution double loop A/AP sites destabilized the wild-type A(htel-21) GQ (*T*_*m*_ 66.1°C) by 6–8°C, whereas, when each of the three TTA loops contained an A/AP or one TTA loop was replaced by three A/AP sites, the wild type was even moderately stabilized, by 1-2°C. The unmodified GQ of TA(htel-21)TT (*T*_*m*_: 62.0°C) was extensively destabilized by single tetrad G/APs, by 15 to 36°C; the latter figure belonged to the middle tetrad substitutions of the three-tetrad GQ. Depending on its position, the single A/APs diminished or intensified the damaging effect of G/APs. The largest negative effects were observed when the G/AP was in the middle tetrad: only partially folded structures were formed at 37°C. Half of the 21 variants of double and triple clustered G/AP sites led to more or less unfolded structures at 37°C; others had *T*_*m*_s between 31 and 37°C, meaning partial folding only. Three of those oligodeoxynucleotides containing one G/AP in the middle tetrad remained unstructured. The single to triple A/APs in the loops increased the population of parallel strands in their structures, thus shifting the antiparallel A(htel-21) GQs into hybrid or parallel forms. Single G/APs and thus also the G/AP plus A/AP combinations, however, inhibited the formation of parallel strands and these GQs adopted antiparallel topologies. CD spectra matched those of the Na^+^-stabilized htel GQs. The* in vitro* results may suggest that formation of clustered lesions in the chromosome-capping structure, in the Shelterin complex, can induce unfolding of existing GQ structures, which, in turn, can lead to telomere shortening [342].

### 5.3. Epigenetic Modifications

*5-Methyl- and 5-Hydroxymethylcytosines (m* ^5^ *C and hm* ^5^ *C)*. Epigenetic 5-methylation of cytosine is among the processes that control the gene expression in human cells, which has also associations with the development of cancer and other diseases [343–345]. Methylation usually occurs at CpG sites and 70–80% of the CpG sites in mammalian cells are methylated. m^5^C accounts for ~1% of all DNA bases in the human genome [344, 346]. The methylation is a reversible enzymatic process [347]. Demethylation of m^5^C (Figure 16,** 2**) follows stepwise through 5-hydroxymethylcytosine (hm^5^C) (Figure 16,** 3**), to 5-formylcytosine and finally to 5-carboxylcytosine, and this base is then excised by the subsequent repair processes. Interestingly, all these bases have been found to occur in mammalian embryonic stem (ES) cells [348], indicating that the modified cytosines are stable and may themselves have some role in gene regulation. Their abundance in cells is very low, ~0.5%, ~0.002%, and 0.0003%, respectively, for the three oxidized derivatives of m^5^C in mouse ES cells [349]. Their level also depends on age, at least in the brain [350]. Regarding the presence of m^5^C in CpG sites of GQs, the P1 promoter sequence of bcl-2 oncogene, the CG_3_CGCG_3_AG_2_A_2_G_5_CG_3_AGC was first examined, where the cytosines at C1, C5, C7, and C21 positions were methylated. The methylations induced the formation of GQ in the P1 promoter by greatly increasing the thermal stability of the structure. Methylation also protected the GQ from unfolding by the complementary strand. The proposed mechanism explained the transcription inactivation by hypermethylation that induces formation of GQ from duplex in the promoter and the gene activation by hypomethylation [351]. The 5-hydroxymethylated cytosines (hm^5^C) are also involved in the regulation of gene expression of certain genes. The studies described by Molnar et al. [352] characterized the effects of hm^5^C-modified CpG islands on the GQ (and the i-motif) structures. Single hm^5^C was incorporated into the G-rich (and C-rich) sequences from the vascular endothelial growth factor (VEGF) promoter. The results indicated that two of the three hm^5^C-containing loops showed a significant decrease in thermal stability of the GQ (on the contrary, thermal stability of the i-motif increased somewhat by the hm^5^C) [352]. Another study was carried out with the G-rich and C-rich strand models of the C9orf72 repeat, the (GGGGCC)_8_ and (GGCCCC)_8_ DNA oligonucleotides, to investigate the effect of epigenetic signals on the macromolecular structure. Each C was replaced by m^5^C or hm^5^C at each CpG motive. The G-rich strands formed G-tetrad- and various mixed G + C tetrad-containing unusually long intramolecular chair-type antiparallel GQs. GQs of the C-rich strands contained C tetrads and also mixed G + C tetrads while no i-motif was observed. The m^5^C moderately increased, by 1.2°C, while hm^5^C considerably decreased, by 4.7°C, the thermal stability of the unmodified intramolecular GQ of the G-rich strand in K^+^ [353].

*8-Oxoguanine (o* ^8^ *G)*. ROS, the reactive forms of oxygen, are free radicals that have unpaired electrons in their outer orbits. ROS can cause significant damage in cells, where DNA is one of the major targets. Recent studies, however, also demonstrated that ROS have normal physiological functions as well. For instance, o^8^G (Figure 15,** 1**), one of the main initial damage products of ROS, has recently been described by Fleming et al. [354] as it is also an epigenetic modification in gene promoters that regulate the transcription via the repair process of the base excision repair (BER). Gene expression was found to occur when o^8^G is formed in the G-rich, potentially GQ-forming DNA sequences. The damage initiated the action of the BER by the 8-oxo-2′-deoxyguanosine DNA glycosylase (hOGG1), whose reaction yields an abasic (AP) site. The AP site enabled unwinding (melting) of the duplex DNA that facilitated the conversion of the G-rich strand to a GQ fold. These reactions finally resulted in the activation of the VEGF or NTHL1 genes. The authors identified 61 human DNA repair genes that might be activated by this mechanism [354]. Later, Fleming and Burrows have confirmed these findings and also compared the epigenetic pathway of o^8^G with that of another base analog, the 5-methylcytosine [355].

## 6. Concluding Remarks

This study illustrates the established, widespread use of synthetic nucleotide analogs in the structural studies of the most researched noncanonical nucleic acid structures, the GQs. Application of the discussed, known analogs and introduction of new derivatives is not going to stop; thus we can expect further growth of the field. Significant advances can be anticipated in the elucidation of the effect of numerous, unexplored single and clustered natural base lesions on the structures and repair of GQs. Recent findings of epigenetics on the role of the formation of some oxidative DNA base lesions followed by conformation switching of the G-rich duplex DNAs to GQs suggest the opening of a new area in the gene expression research.

## Abbreviations

GQ: G-quadruplex
*T*_*m*_: Half-way temperature of the thermal unfolding profile of the GQs
htel: Human telomere repeat sequences
TBA: Thrombin-binding aptamer
CD: Circular dichroism.

## Additional Points

In most cases, DNA GQs are discussed here, and therefore the “d” is omitted from the sequence; for instance, d(TGGGAG) is denoted as TGGGAG, or the htel DNA, such as the d[G_3_(TTAG_3_)_3_], is denoted as G_3_(TTAG_3_)_3_ or in an even shorter way as htel-21. The 5′- or 3′-extended htel DNA oligonucleotides of the basic 21-nucleotide-long (21-mer) htel-21 are also abbreviated as, for example, TA(htel-21)TT that refers to d[TAG_3_(TTAG_3_)_3_TT]. The bimolecular and tetramolecular GQs are abbreviated by using square brackets, such as with [TGGGT]_4_. RNA GQ sequences are abbreviated with small letters, like ugggu.
